# Comprehensive analysis of *CTNNB1* in adrenocortical carcinomas: Identification of novel mutations and correlation to survival

**DOI:** 10.1038/s41598-018-26799-2

**Published:** 2018-06-05

**Authors:** Rajani Maharjan, Samuel Backman, Tobias Åkerström, Per Hellman, Peyman Björklund

**Affiliations:** Department of Surgical Sciences, Uppsala University, Uppsala, Sweden

## Abstract

The Wnt/β-Catenin signaling pathway is one of the most frequently altered pathways in adrenocortical carcinomas (ACCs). The aim of this study was to investigate the status of Wnt/β-Catenin signaling pathway by analyzing the expression level of β-Catenin and the mutational status of *APC*, *AXIN2*, *CTNNB1*, and *ZNRF3* in ACCs. Mutations in *APC*, *CTNNB1*, *ZNRF3* and homozygous deletions in *ZNRF3* were observed in 3.8% (2/52), 11.5% (6/52), 1.9% (1/52) and 17.3% (9/52) of the cohort respectively. Novel interstitial deletions in *CTNNB1* spanning intron 1 to exon 3/intron 3 were also found in 7.7% (4/52) of the tumours. All the observed alterations were mutually exclusive. Nuclear accumulation of β-Catenin, increased expression of Cyclin D1 and significantly higher expression of AXIN2 (p = 0.0039), ZNRF3 (p = 0.0032) and LEF1(p = 0.0090) observed in the tumours harbouring the deletion in comparison to tumours without *CTNNB1* mutation demonstrates that the truncated β-Catenin is functionally active and erroneously activates the downstream targets. Significantly lower overall survival rate in patients with tumours harbouring alterations in *APC*/*CTNNB1*/*ZNRF3* in comparison to those without mutation was observed. In conclusion, the discovery of novel large deletions in addition to the point mutations in *CTNNB1* infers that activation of Wnt/β-Catenin pathway via alterations in *CTNNB1* occurs frequently in ACCs. We also confirm that alterations in Wnt/β-Catenin signaling pathway members have a negative effect on overall survival of patients.

## Introduction

The Wnt/β-Catenin signaling pathway is vital for adrenal development, differentiation, and cell renewal^1^. In the absence of Wnt signaling, β-Catenin bound by the destruction complex (comprising Axin, Apc, CK1, and GSK3β) is phosphorylated by CK1 and GSK3β on its serine/threonine residues. This leads to ubiquitin-mediated proteolysis of β-Catenin. However, in the presence of Wnt signaling, Wnt ligands bind to their FZD/LRP receptors leading to recruitment of destruction complex to the plasma membrane followed by phosphorylation of GSK3β. This interferes with the destruction complex’s activity and promotes stabilization and accumulation of newly synthesized β-Catenin with subsequent transcription activation or inhibition of the downstream target genes^2^. Recently R-spondin and ZNRF3/ RNF43 module has been recognized as an upstream regulator of Wnt/ β-Catenin signaling pathway^3^. ZNRF3/ RNF43 are members of E3 ubiquitin ligase family and have been found to reduce the Wnt signaling by ubiquitination-mediated degradation of Wnt receptors, Frizzled. In the presence of R-spondin (a Wnt signaling agonist), however, ZNRF3/RNF43 are cleared from the membrane which eventually leads to accumulation of Wnt receptors and increased Wnt signaling.

Structurally, β-Catenin largely consists of three domains; the N-terminal domain, central armadillo repeats and the C-terminal domain. The central region (residues 141–664) consists of 12 armadillo repeats and is the most conserved region of β-Catenin harbouring binding sites for interaction with other proteins such as APC, Axin, and TCF^4–6^. The N-terminal domain harbours serine/threonine residues which serve as GSK3β and CK1-mediated phosphorylation sites, and are recognized by β-TrCP ubiquitin ligase^7–9^. Mutations in exon 3 of the *CTNNB1* gene (encoding β-Catenin) causing loss of serine/threonine residues are the most frequent mechanism of an erroneously activated Wnt/β-Catenin signaling pathway^10–12^. Sporadic cases of deletions and insertions in exon 3 and deletion of the entire exon 3 have also been observed^11,13^.

Aberrations in Wnt/β-Catenin signaling through activating mutations of *CTNNB1* are known to occur in different types of adrenocortical adenomas and cancers^14,15^. Adrenocortical carcinoma (ACC) is a rare type of malignancy with an incidence of 1–2 per million^16^. *CTNNB1* mutations have been observed in approximately 10–15% of ACCs^17–20^, although aberrant activation of Wnt/β-Catenin signaling is observed in a much higher proportion of ACC tumours, most of them are not caused by known *CTNNB1* mutations^11,21^. Recent pan-genomic studies of ACCs have shown that alterations in other members of Wnt/β-Catenin signaling pathway such as *ZNRF3* and *APC* could also lead to activation of the pathway^18,19^. Furthermore, benign adrenal tumours with aberrant β-Catenin nuclear accumulation have been associated with a larger size and malignant adrenal tumours with aberrant β-Catenin nuclear accumulation defined poor prognosis^11,21^.

Here we have analyzed the mutation status of *APC*, *AXIN2*, *CTNNB1* and *ZNRF3* and expression of β-Catenin in a large cohort of ACCs. We also describe novel interstitial deletions in *CTNNB1* and their functional impact in ACCs.

## Results

### Mutation detection and *in silico* analysis

We have screened 61 tumours originating from 52 patients for mutations in members of Wnt/β-catenin signaling pathway; *APC*, *AXIN2*, *CTNNB1*, and *ZNRF3*. Mutations in *APC*, *CTNNB1*, and *ZNRF3* genes were found in two, six and one sample respectively whereas none of the analyzed samples harboured any mutations in *AXIN2*. One of the *APC* mutations was germline, c.4666_4667insA (p.T1556NfsTer3), and the other was a somatic mutation, c.4391_4394del (p.E1464GfsTer8) (Supplementary Fig. S1). Both mutations cause a frameshift creating a premature stop codon. The patient with the tumour harbouring somatic mutation, c.4391_4394del, also harboured a 16.6 kb large germline duplication spanning over exon 2 and 3. Missense mutations in exon 3 of *CTNNB1*, c.133T > C (p.S45P), c.104T > A (p.I35N), c.136C > A (p.S45Y), c.109C > G (p.S37C), c.105G > A (p.G34R) and c.134C > T (p.S45F) were found in 6 individual tumours (Supplementary Fig. S1), whereas exon 5 was devoid of any mutation. All the observed mutations in *CTNNB1* occurred in established hotspot residues. A novel missense mutation, c.646C > T (p.H216Y), was found in *ZNRF3* (Supplementary Fig. S1). All the observed mutations were mutually exclusive. All the affected residues by missense mutations were conserved across the investigated species (Supplementary Fig. S2), and *in silico* analysis using Polyphen-2, SIFT and PROVEAN predicted these mutations as deleterious (Supplementary Table S1). Allele comparison of the SNP (rs11564435) in *CTNNB1* found in our cohort showed similar allele frequency (χ^2^ = 2.0517, P = 0.1520) to that of European subpopulations^22^.

### Whole Genome Sequencing identifies interstitial deletions

cDNA-specific PCR amplification using primers spanning exon 1 to 6 of *CTNNB1* was performed on cDNA of all 61 ACC tumours to identify deletions spanning exon 3. Visualization of the products through gel electrophoresis revealed presence of shorter bands in four tumours (ACC 18, ACC 21, ACC 37 and ACC 40) of approximately 630 bp in size along with a 920 bp apparently wild-type product (Supplementary Fig. S3a). Sanger sequencing of gel-excised and purified products revealed a mutation r.-48_241del leading to deletion of the entire exon 2 and 3 (here forth called ∆(2 + 3) deletion) in all four samples (Supplementary Fig. S3b).

**Figure 1:**
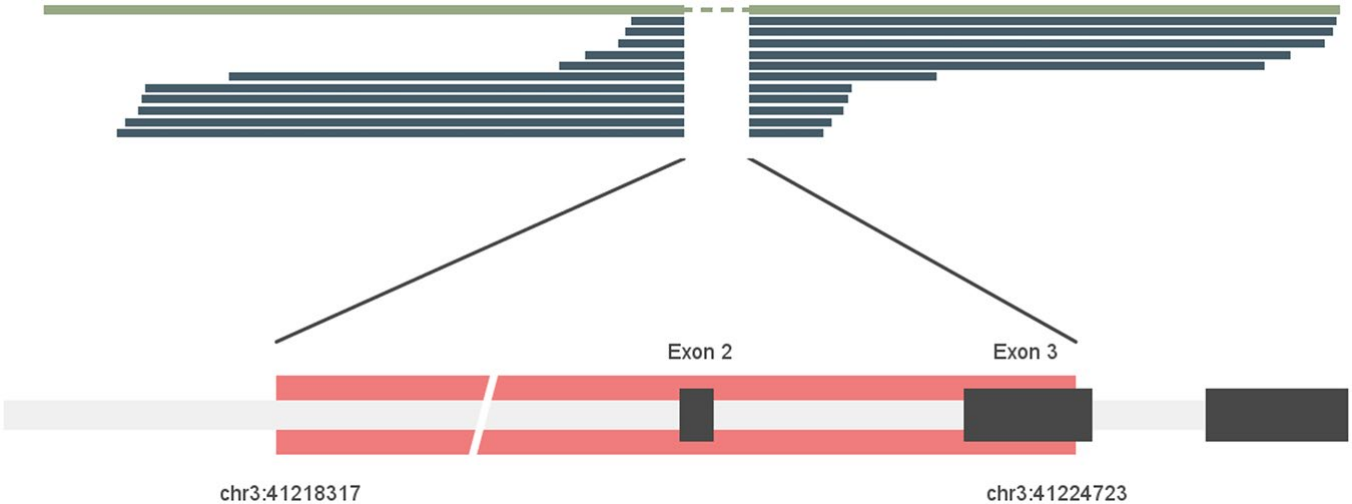
Interstitial deletion detected by whole genome sequencing. Eleven reads (dark green) overlapping the deleted region aligned to the reference sequence show deletion from chr3:41218317 to chr3:41224723 spanning over intron 1 to exon 3 of *CTNNB1* for sample ACC40.

**Figure 2:**
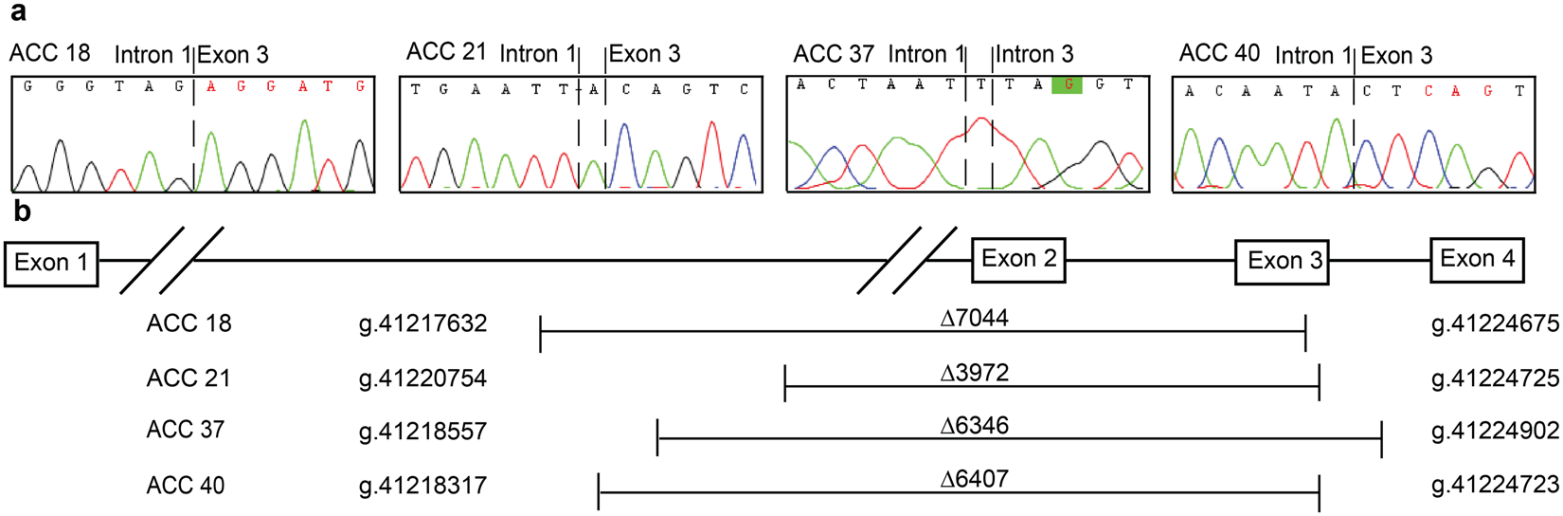
(**a**) Electropherograms showing deletion junctions. Sanger sequencing spanning intron 1 to exon 4 of ACC 18, ACC 21, ACC 37 and ACC 40 shows deletions g.41217632-g.41224675del, g. 41220754_g.41224725delinsA, g. 41218557_g. 41224902delinsT, g.41218317_g.41224723del.(**b**) Map of deletions for ACC 18, ACC 21, ACC 37 and ACC 40 harbouring deletion of ∆7044, ∆3972, ∆6346, and ∆6407 base pairs spanning exon 1 to exon3/intron 3.

Exclusion of exons in the mRNA may occur due to several mechanisms such as alternative splicing, genomic deletions etc. If such exclusion was caused by genomic deletion, the exclusion of exon 2 and 3 of *CTNNB1* could be caused by deletions occurring anywhere from intron 1 to intron 3. Due to the large size of this genomic region (intron 1 spans over 24,350 bp); we performed whole genome sequencing in one tumour harbouring the deletion (ACC40). We found a deletion of 6407 bp (g.41218317-g.41224723del) spanning over intron 1 to exon 3 (Fig. 1) in 11 of the reads covering the deleted region. We verified the result by Sanger sequencing in ACC40 as well as in the other three tumours using sets of primers flanking the deleted region (Fig. 2a and Supplementary Table S2). The other three tumours, ACC18, 21 and 37, harboured deletion mutations g.41217632_g.41224675del (∆7044 bp), g.41220754_g.41224725delinsA (∆3972 bp) and g.41218557_g. 41224902delinsT (∆6346 bp), respectively (Fig. 2a and Supplementary Table S3). The deletions in all four tumours occurred at overlapping loci (Fig. 2b and Supplementary Table S3). Interestingly, we observed presence of sequence microhomology or reverse complement sequence at the deletion breakpoint of all four tumours harbouring these interstitial deletions (Supplementary Tables S4 and S5). In addition, evaluation of sequences 50 bp upstream and downstream of the deletion start site showed that all four deletions occurred at AT-rich (≥70%) sites (Supplementary Table S6).

### Expression of truncated β-Catenin and Wnt/β-Catenin downstream targets

**Figure 3:**
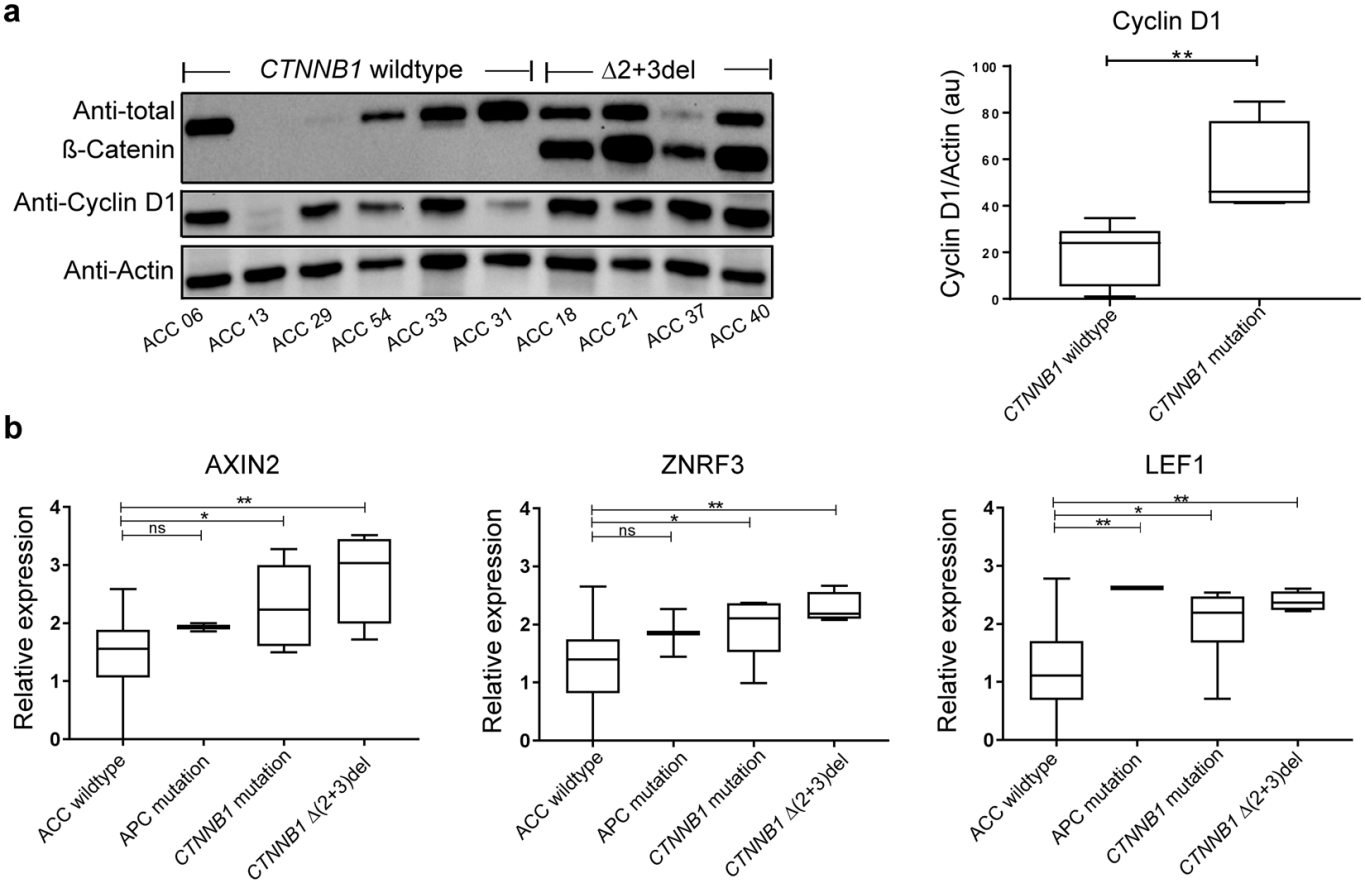
(**a**) Western blot results showing expression of wildtype and truncated β-catenin, Cyclin D1, and Actin in all four samples harbouring deletions in comparison to samples without *CTNNB1* mutation or deletion with quantification of Cyclin D1expression. The protein lysate was prepared from the tumours, subjected to SDS page followed by western blot with anti-β-catenin, anti-Cyclin D1and anti-Actin. Uncropped original blots are shown in Supplementary Fig. 5. (**b**) mRNA expression of AXIN2, ZNRF3 and LEF1 in tumour samples harbouring *APC* mutation, *CTNNB1* mutation and ∆(2 + 3) deletion in comparison to rest of the cohort (p values for AXIN2 and ZNRF3 in ACC wildtype vs *APC* mutation are 0.1925 and 0.2598 respectively).

Deletion of exon 2 and 3 eliminates the natural transcription start site of *CTNNB1*. In order to investigate the functional impact of the ∆(2 + 3) deletion on the protein product, β-Catenin, we performed Western blot analysis on the tumour protein lysates, which demonstrated expression of wildtype as well as truncated protein in samples harbouring the ∆(2 + 3) deletion (Fig. 3a). Exclusion of exon 2 and 3 leads to partial deletion of the N-terminal, which harbours GSK3β, CK1 phosphorylation and β-TrCP recognition sites (Supplementary Fig S4). Higher accumulation of the truncated β-Catenin in comparison to wildtype was observed in all four samples with the deletion (Fig. 3a).

Analysis of Cyclin D1 protein expression showed significantly higher expression (p = 0.0095) in the samples with the deletion in comparison to analyzed tumours without *CTNNB1* mutation (Fig. 3a and Supplementary Fig. S5). mRNA expression analyses revealed high expression levels of AXIN2 and LEF1 in samples harbouring *CTNNB1* mutations and deletions. The expression of AXIN2 and LEF1 was significantly higher in tumours harbouring a ∆(2 + 3) deletion in comparison to those without mutations in analyzed genes (ACC wildtype (n = 39) vs ∆(2 + 3) deletion (n = 4), p = 0.0039 and p = 0.0090 respectively) and was comparable to the tumours harbouring *CTNNB1* missense mutations (ACC wildtype (n = 39) vs *CTNNB1* mutants (n = 5), p = 0.0211 and p = 0.0451 respectively; Fig. 3b). Expression levels of ZNRF3 mRNA were significantly higher in tumours harbouring the ∆(2 + 3) deletion (p = 0.0032) and missense mutations (p = 0.0210) in comparison to those without *CTNNB1* mutations (Fig. 3b). A trend towards higher expression of Cyclin D1 mRNA was observed in samples harbouring *CTNNB1* mutations and deletions in comparison to those without but did not reach statistical significance (Supplementary Fig. S6).

### Copy number variation Analysis

GISTIC analysis of SNP array data (n = 52) showed significant deletions occurring at *ZNRF3* locus (q < 0.01). Homozygous deletions at *ZNRF3* locus were observed in 9/52 tumours. Analysis of SNP array data from recurrent tumours available for 4/9 cases with *ZNRF3* deletion in the primary tumour showed presence of *ZNRF3* deletion in all four recurrent tumours. The deletions found in *ZNRF3* were mutually exclusive to *APC*, *CTNNB1* and *ZNRF3* mutations. Samples with *ZNRF3* deletions showed significantly lower expression of ZNRF3 (p = 0.0001) and higher expression of AXIN2 (p = 0.0274) and LEF1 (0.0081) in comparison to the samples devoid of mutations in investigated genes (Supplementary Fig. S7). Copy number variation status at the *CTNNB1* locus was also analyzed. In ACCs without any *CTNNB1* mutation (n = 51), 15 samples harboured loss, nine harboured gain, 11 harboured copy neutral loss of heterozygosity (cnLOH), five harboured loss with allelic imbalance whereas 11 were diploid at the *CTNNB1* locus 3p22.1 (Supplementary Fig. S8a). Amongst tumours with *CTNNB1* missense mutation, one harboured gain and three harboured cnLOH. Out of the four tumours harbouring a *CTNNB1* ∆(2 + 3) deletion, two harboured gain and one harboured cnLOH (Supplementary Fig. S8b). Analysis of β-Catenin expression in relation to the copy number status did not show any correlation.

### β-Catenin expression in adrenocortical carcinomas

Active β-Catenin expression status was assessed by Western blot analysis for all the available primary tumours (n = 50). Active β-Catenin expression was detectable in 31/50 analyzed tumours (Supplementary Fig. S9). Both tumours with APC mutation and all six tumours harbouring β-Catenin missense mutations showed expression of active β-Catenin at varied levels. The anti-active β-Catenin antibody detects the unphosphorylated serine/threonine residues in exon 3. Hence quantification of active β-Catenin for four tumours harbouring the ∆(2 + 3) deletion was not possible due to the lack of epitope. One of these four tumours (ACC21), however, had a detectable level of wildtype active β-Catenin. Amongst the analyzed samples harbouring *ZNRF3* alterations (8 deletions and 1 mutation), only three samples showed expression of the active β-Catenin.

**Figure 4:**
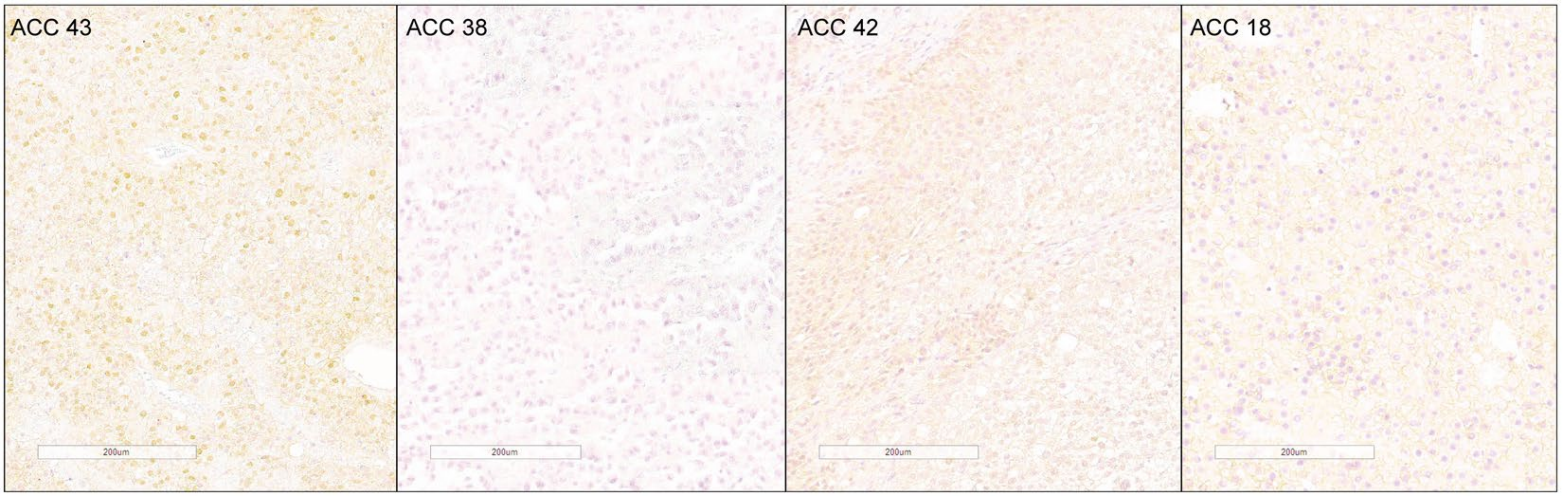
Representative photomicrographs showing different groups of ACC in terms of β-catenin nuclear expression and *CTNNB1* mutation status. ACC 43, a tumour with *CTNNB1* mutation and β-catenin nuclear accumulation; ACC 38, a tumour without *CTNNB1* mutation showing lack of β-catenin nuclear accumulation; ACC 42, a tumour without *CTNNB1* mutation with nuclear accumulation of β-catenin and ACC 18, a tumour with *CTNNB1* mutation and absence of nuclear accumulation of β-catenin.

Expression of β-Catenin was also assessed by immunohistochemistry on primary tumours (n = 49), this revealed cytoplasmic expression in 45 tumours and nuclear expression in 25 tumours (Supplementary Fig. S10). Nuclear expression of β-Catenin was observed in all six tumours harbouring β-Catenin missense mutations and in 3/4 tumours harbouring the ∆(2 + 3) deletion (Supplementary Table S7). Hence on the basis of β-Catenin mutation and corresponding nuclear expression status, the tumours could be divided into tumours (1) with *CTNNB1* mutation and nuclear expression (n = 9), (2) with *CTNNB1* mutation but without nuclear expression (n = 1), (3) with wildtype *CTNNB1* and without nuclear expression (n = 23) and (4) with wildtype *CTNNB1* and nuclear expression (n = 16) (Figs 4 and S10 and S11). Amongst 16 *CTNNB1* wildtype tumours with the presence of nuclear accumulation, five harboured *ZNRF3* deletion and one harboured *APC* mutation.

Correlative analyses of nuclear accumulation and active β-Catenin expression were performed on 44 primary tumours analyzed by both methods (Supplementary Fig. S12). Nuclear expression and presence of active β-Catenin correlated for 18 tumours. Expression status did not correlate for the remaining 13 tumours. Ten tumours with expression of the active β-Catenin lacked nuclear expression whereas the remaining 3 tumours with nuclear expression of β-Catenin did not show accumulation of the active β-Catenin.

### Survival and clinical characteristics

Clinical details of the patients included in this study are listed in Supplementary Table S7. A marked female predominance (F/M ratio = 1.6) was present. Median age at operation and tumour size of the cohort was 52 years and 10 cm respectively. Univariate analysis on available clinical criteria (age, ENSAT staging (I/II vs II/IV), gender, cortisol production, tumour size) showed a significant prognostic value of ENSAT staging in our cohort (Supplementary Table S9) (HR = 4.076, P = 0.001). The significance of its prognostic value remained after removal of childhood ACCs (<15 yrs, HR = 4.160, P = 0.001). Median age at operation and tumour size for the group of tumours with APC/*CTNNB1/ZNRF3* alterations were 52.5 years and 10 cm respectively and were not significantly different from those without mutations (46 yrs, p = 0.3517, and 10 cm, p = 0.5731; Supplementary Fig. S13a,b). There were no significant differences in any analyzed clinical parameters between groups of tumours with and without *APC/CTNNB1/ZNRF3* alterations, nuclear β-Catenin expression or active β-Catenin expression (Supplemental Table S8).

**Figure 5:**
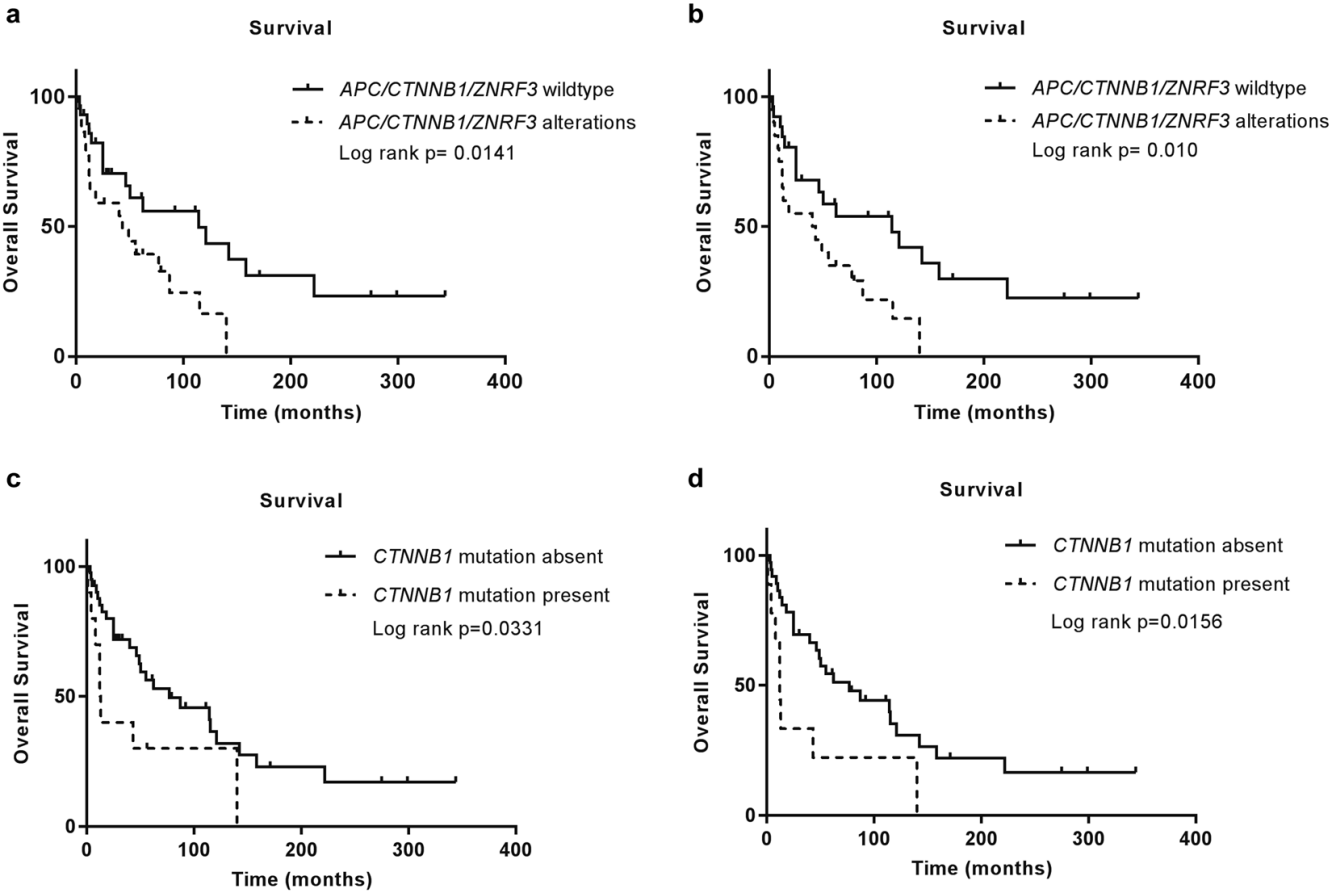
Overall survival rates for (**a**) patients with tumours harbouring *APC/CTNNB1/ZNRF3* alterations in comparison to ones without, (**b**) overall survival remained significant after removal of patients under 15years of age at the time of operation. (**c**) Overall survival rates for patients with tumours harbouring *CTNNB1* mutations and deletions in comparison to ones without, (**d**) overall survival in chart c remained significant after removal of patients under 15 years of age at the time of operation.

The median overall survival of the cohort was 62 months. Survival was compared based on presence or absence of *APC*/*CTNNB1/ZNRF3* alterations. The median overall survival rate for patients with a tumour harbouring *APC*/*CTNNB1/ZNRF3* alteration was significantly lower compared to the ones without (43 vs 114 months, log rank p = 0.0141, Fig. 5a). The significance remained after removal of childhood ACC cases (41.5 vs 114 months, log rank p = 0.010, Fig. 5a). Disease-free survival was not significantly different between the group of patients with and without *APC*/*CTNNB1/ZNRF3* alterations (22 vs 48 months, log rank p = 0.0.1152, Supplementary Fig. S14a). Univariate analysis on the cohort based on the mutational status of *APC*/*CTNNB1/ZNRF3* also showed a significant prognostic value (Supplementary Table S9) in the entire cohort (HR = 2.493, P = 0.013) as well as in adult ACCs (HR = 2.481, P = 0.014). However, the prognostic value of *APC*/*CTNNB1/ZNRF3* mutation did not reach statistical significance in the multivariate analysis with ENSAT staging (Supplementary Table S9).

The median overall survival rate for patients with tumours harbouring *CTNNB1* mutations and deletions alone was significantly lower compared to the ones without (12.5 vs 77 months, log rank p = 0.0331, Fig. 5b). The difference of survival rate remained significant after removal of childhood ACC cases (12 vs 77months, log rank p = 0.0156, Fig. 5b). The difference in disease-free survival between the group of patients with and without *CTNNB1* mutations alone did not reach statistical significance (8 vs 41 months, log rank p = 0.1148, Supplementary Fig. S14b). A trend towards lower median overall survival rate for patients with tumour showing nuclear accumulation of β-Catenin was observed (25 vs 87 months in the entire cohort and 18 vs 87 months in adult ACC), however, it did not reach statistical significance (Supplementary Fig. S15).

## Discussion

Mutations and deletions in genes involved in the Wnt/β-Catenin signaling pathway are observed in approximately 40% of ACCs and mutations in *CTNNB1* alone accounts for up to 15% of studied tumours^18,19^. Missense mutations or deletions in/of exon 3 affecting serine/threonine phosphorylation sites of β-Catenin have been observed in these types of tumours and were observed in 11.5% of our cohort. We show frequent (4/52, 7.7%) novel interstitial deletion, ∆(2 + 3) deletion, in *CTNNB1* leading to expression of a truncated protein. To our knowledge, such interstitial deletions leading to skipping of exon 2 and 3 have not been reported in adrenal tumours. Deletion of exon 2 and 3 in *CTNNB1* was previously observed in one melanoma cell line^23^ and in one malignant melanoma^23,24^. Such deletion of a whole exon could occur due to several phenomena such as interstitial deletions at the genomic level, alternative splicing or other post-translational processing. However, in both of the studies the mutation was observed in and limited to cDNA derived from the specimens and hence was not conclusive regarding the cause of the deletion.

Whole genome sequencing allowed us to map interstitial deletions spanning intron 1 to exon 3/intron3 of *CTNNB1*. Interestingly, deletions occurred at similar regions in all four tumours leading to deletion of exon 2 and either whole (n = 1/4) or fractions of exon 3 (n = 3/4). Despite deletion of exon 2 carrying the transcription start site, and complete exclusion of exon 3, expression of an in-frame truncated protein was observed in all four tumours. This implicates that for the production of the in-frame protein product, the transcription and translation machinery skips the remainder of exon 3 and starts translation from another in-frame start codon. Generally, an exon can harbour an exonic splicing enhancer (ESE) or an exonic splicing silencer (ESS) element^25^. ESE is required for the efficient splicing and inclusion of the exon it resides in whereas ESS is required for skipping. Alterations in ESE elements are known to cause either inefficient splicing, leading to partial or complete exclusion of the exon^26,27^. This and several other types of nonsense-mediated alternative splicing mechanisms could explain the complete exclusion of exon 3 in these tumours^27^.

β-Catenin turnover relies on the presence of its serine/threonine rich N-terminal region that becomes phosphorylated by GSK3β and CK1^3,7,28^. Deletions or mutations in this region cause stabilization and accumulation of β-Catenin^23^. Constitutively active β-Catenin occurring via deletion of exon 3 alone was shown to induce adrenal hyperplasia in female mice, which had advanced to cancer in some cases^29^. N-terminal deleted β-Catenin with intact highly conserved armadillo repeats important for its structural and functional activity^30^, accumulates in the cytoplasm and nucleus^31^ and mimics activated Wnt signaling pathway in *Xenopus* and *Drosophila*^28,32^. The observed ∆(2 + 3) deletion in β-Catenin in our study causes a removal of first 87 amino acids given that the first in-frame start site in exon 4 is utilized. Such N-terminally deleted β-Catenin, lacking the first 86 and 89 amino acids (∆N86 and ∆N89 β-Catenin) was shown to cause increased LEF1 mediated transcription *in vitro* and increased proliferation, apoptosis and decreased migration in mouse intestinal epithelial cells^31,33^. ∆N89 β-Catenin expression in mouse mammary glands was shown to cause development of aggressive adenocarcinomas in 100% of the studied female mice^34^. These observations provide an excellent model for ∆(2 + 3) deletion effects.

Immunohistochemical analysis of the tumours with mutant β-Catenin showed nuclear and cytoplasmic accumulation of β-Catenin in all but one tumour with the ∆(2 + 3) deletion. An absence of nuclear accumulation of β-Catenin in adrenocortical tumours harbouring activating β-Catenin mutations has been observed before^11,35^. Activation of Wnt/β-Catenin signaling increases the expression of ZNRF3^3^, LEF1, Cyclin D1^36^ and AXIN2, which are direct targets of β-Catenin^29,37,38^. Similar to *AXIN2*, *ZNRF3*, a newly added member of the Wnt/β-Catenin signaling pathway, is a negative regulator of the pathway^3^. *ZNRF3* is also the most frequently deleted gene in ACCs^18,19^ and is reported to be upregulated in colorectal tumours exhibiting activated Wnt/β-Catenin signaling pathway^3^. Our findings of high expression of Cyclin D1 and significantly higher expression of AXIN2, LEF1 and ZNRF3 mRNA in tumours harbouring the ∆(2 + 3) deletion at a similar level to those with hot spot mutations suggest the activating nature of these deleterious mutations in ACCs.

Mutations in members of Wnt/β-Catenin signaling pathway and nuclear accumulation of β-Catenin are correlated with poor survival^21^. Association of *CTNNB1* mutation with low survival observed in our cohort confirms and validates the previous observations. In our study, the association was even more evident in the group of patients with tumour harbouring alterations in *APC*/*CTNNB1*/*ZNRF3*.

While aberrations in Wnt/β-Catenin signaling pathway and nuclear accumulation of β-Catenin are observed with similar frequency (40%) in ACCs, the presence of aberrancy (*CTNNB1* missense mutation and *ZNRF3* deletion) and accumulation of β-Catenin do not always occur together^11,20,35^. In addition to such observation in our study, we also observed that tumour samples with the accumulation of nuclear β-Catenin did not always show accumulation of active β-Catenin and vice versa. Tumour heterogeneity and complexity of malignant tumours like a culmination of whole genome hypo- or hyperploidy, accumulation of mutations and alterations in methylation, RNA expression and miRNA expression which is also observed in ACCs might lead to different phenotypic observations in tumours than expected by just a presence of the single mutations.

The interstitial deletion in *CTNNB1*, as well as the alterations in *APC* and *ZNRF3*, observed in our cohort adds to the spectrum of aberrancies occurring in ACCs with activated Wnt/β-Catenin pathway. Further, investigation of mechanisms leading to interstitial deletions in *CTNNB1* is warranted.

## Materials and Methods

### Patients

Sixty-one tumours from 52 patients (20 males, 32 females; mean age 46.7 ± 20.3 years) were operated for adrenocortical cancer (ACC), with adrenalectomy. Amongst 52, five cases were childhood ACC (Supplementary Table S8). The tumour size (largest diameter) ranged from 5–30 cm (mean 12 ± 6.1 cm). The ENSAT score^39^ is presented in Supplementary Table S8. Patient charts were scrutinized to establish survival data. Cortisol excess was diagnosed by clinical and biochemical evaluation, including 24- hour urinary cortisol and/or midnight cortisol and early-morning plasma adrenocorticotropic hormone (ACTH), in some cases 1 mg dexamethasone suppression test was performed. Other hormone excesses were determined based on clinical signs and appropriate blood hormone assays according to routine diagnostic procedures at Uppsala University Hospital. Patients who had metastases at the time of surgery for a primary tumour were excluded from disease-free survival analysis.

### Tumour samples

All the tumours included in the study were obtained from patients operated at Uppsala University hospital and were handled according to institutional guidelines and regulations. The tumours were snap frozen in liquid nitrogen and stored at −70 °C. Tumours were also formalin fixed for 24 hours and then paraffin embedded for preparation of formalin-fixed paraffin embedded (FFPE) tissue samples. All biologic specimens were obtained from Uppsala Biobank, Endocrine tumour collection [Ethical approval 00-128/3.15.2000, Local ethical vetting board in Uppsala (Regionala etikprövningsnämnden i Uppsala)]. The study was approved by the regional ethical review board in Uppsala [11-375/1.1.2011, Local ethical vetting board in Uppsala (Regionala etikprövningsnämnden i Uppsala)]. Ethical approval and informed consent from all the included patients (from guardians if younger than 18) were obtained prior to the study.

### DNA/RNA extraction and cDNA synthesis

Tumours were cryosectioned and hematoxylin-eosin stained to assure >80% tumour content. When necessary, after histological analysis, tumours were macro-dissected, to achieve maximum tumour cell content. DNA and RNA were prepared from cryosectioned tumour samples and matching normal tissue using Allprep DNA and RNA kit (Qiagen, Hilden, Germany). Blood DNA was prepared using Blood and Tissue kit (Qiagen, Hilden, Germany). Quality controlled RNA was used to prepare cDNA using RevertAid First strand cDNA Synthesis kit (ThermoFisher Scientific, MA, USA).

### PCR amplification and qPCR

Specific primers for DNA and cDNA were used to amplify the regions of interest (Supplementary Table S10). Primers for *APC* and *AXIN2* were described before^21,40^. Obtained PCR products were visualized using 1.5% Agarose gel with SYBR green. The PCR products were gel excised when necessary and purified using PureLink Quick Gel Extraction Kit (cat#K210012, Thermo Fisher Scientific, MA, USA). PCR products were sequenced using automated Sanger sequencing at Beckman Coulter Genomics, UK. The sequence chromatograms were analyzed using Codoncode Aligner 3.7.1 (CodonCode Corporation, MA, U.S) using NM_001904.3 as a reference sequence. Beta-actin was used as a reference gene for qPCR analysis. SsoAdvanced SYBR Green Supermix (Life Science, United States) was used to perform all the qPCRs on a CFX96 Real Time system (Bio-Rad Laboratories, CA, USA). The samples were run in triplicates and the ∆∆CT method was used to determine the relative expression levels. Log-transformed values were used for graphical representation.

### *In Silico* analysis

To analyze the impact of the mutations, the Polyphen-2^41^, PROVEAN^42^ and SIFT^43^ prediction tools were used. Clustal omega, a multiple sequence alignment program, was used to compare the human β-Catenin protein sequence with that of *Danio rerio*, *Mus musculus* and *Xenopus tropicalis*^44,45^.

### SNP array analysis

Tumour DNA was subjected to SNP array analysis using Illumina HumanOmniExpress-Exome-8v (Illumina Inc., CA, USA) at the Science For Life core facility (Uppsala University, Uppsala, Sweden). The raw data were processed with Illumina BeadStudio, and further processed and analyzed using Nexus copy number variation 7.5 software (Biodiscovery, USA). Allele-Specific Copy number Analysis of Tumours (ASCAT) analysis was performed on the SNP array data. The samples were manually re-centered using ASCAT data. Copy number variation analysis was performed using logR ratio and B allele frequency (BAF). For the analysis of frequent deletions occurring in the cohort, the SNP array data was first processed using circular binary segmentation algorithm and were further analyzed using GISTIC tool^46^ in Nexus 7.5.

### Immunohistochemistry

Immunohistochemistry was performed on 4 μm thick FFPE tissue sections: (1) The slides were dried at 60 °C before use (2) sections were treated with xylene, 99.9% ethanol, 90% ethanol, 70% ethanol and dH2O in a series, for paraffin removal and rehydration of the tissue sections (3) sections were treated with hydrogen peroxide (H_2_O_2_) followed by (4) heat treatment using citric buffer for antigen retrieval (5) sections were incubated with normal horse serum (1:10, cat#S-2000, Vector Laboratories), (6) anti- β-Catenin (1:200, cat#sc-376959, Santa Cruz Biotechnology) and (7) secondary antibody (cat#BA-2000, Vector Laboratories) diluted in BSA-PBS buffer (bovine serum albumin and PBS buffer). Chromogenic detection of protein using (8) ABC and (9) DAB (cat#PK-4000 and cat#SK-1000, Vector Laboratories) and (10) nuclear staining using hematoxylin followed. The slides were washed with PBS between each step mentioned above. The sections were then (11) dehydrated by treating them with 70% ethanol, 90% ethanol, 99.9% ethanol and xylene. For frozen tissue immunohistochemistry, 5 μm thick cryosections were generated and (1) fixed in acetone, (2) treated with hydrogen peroxide (H_2_O_2_) and (3) blocked with avidin-biotin. Steps 5, 6, 7, 8, 9 and 10 mentioned above were followed for staining of cryosections.

Normal adrenal tissue was used as a positive control whereas negative controls were obtained by excluding the primary antibody. The samples were scored and categorized as “negative” or “positive” for cytoplasmic staining and as “negative”, “focal positive” (heterogeneous) and “positive” for nuclear staining. For positive cases, the nuclear and cytoplasmic staining intensity was scored manually ascending from 1+ to 5+, independently by two investigators blinded to the mutation status of the investigated genes. Scoring for heterogeneous cases was based on the positively stained sections solely. For graphical representation, the samples with 1+ to 2+ were grouped as ‘low’ and samples with 3+ to 5+ were grouped as ‘high’.

### Whole Genome Sequencing

Extracted DNA was subjected to whole genome sequencing at the SNP&Seq Technology Platform in Uppsala on an Illumina HiSeq. 2500/HiSeqX. The number of generated reads was 667,706,189, out of which 650,970,282 were aligned to the reference resulting in a mean autosomal coverage of 29.67x. The generated reads were aligned to the reference genome (human_g1k_v37) using BWA v. 0.7.12^47^. Following read alignment, duplicate reads were excluded and base quality score recalibration was performed using GATK v. 3.3^48–50^. Insertions, deletions and other structural variants located on chromosome 3 were called on the generated BAM file with Pindel v.0.2.4^51^, using the standard settings and an average insert size of 250 bases. Possible deletion/insertion overlapping the *CTNNB1* locus was manually inspected.

### Western blot

Samples were lysed using cytobuster (Cat#71009, Merk Millipore, MA, USA) supplemented with protease inhibitor cocktail (Roche, Cat#04693124001). Obtained protein lysates were denatured at 95 °C with Laemmli buffer and β-mercaptoethanol for 10 minutes. The heat-treated samples were subjected to SDS-PAGE and transferred to a PVDF membrane. Western blotting was performed on the PVDF membrane as described before^52^ and probed for β-Catenin (1:200, cat#sc-376959, Santa Cruz Biotechnology, TX, USA), active β-Catenin (cat#05–665, Merck Millipore, MA, USA), Cyclin D1 (cat#sc-6281, Santa Cruz Biotechnology, TX, USA) and Actin (cat#sc-1616, Santa Cruz Biotechnology, TX, USA). The expression levels of the protein were normalized using a reference (ACTB) and were quantified using Image Lab software (Bio-Rad Laboratories, CA, USA).

### Statistical analysis

The Mann-Whitney U test was used for quantitative analysis of Cyclin D1 western blot. Kruskal-Wallis test with correction for multiple comparisons by controlling False discovery rate using the two-stage step-up method of Benjamini, Krieger, and Yekutieli was performed for statistical analysis of AXIN2, Cyclin D1, LEF1 and ZNRF3 mRNA expression, utilizing GraphPad Prism 7 (GraphPad Software, CA, USA). Cox proportional hazards regression method was used to determine univariate and multivariate hazard ratios using SPSS software. For survival analysis, the data were censored if the patients were still alive at last follow up. Survival analyses were made using survival curves created by the Kaplan-Meier estimate with the Log-rank test. Statistical significance was determined to be achieved when p < 0.05. The Chi-square tests were used for comparison of nominal data.

## Electronic supplementary material

Supplementary Information
