# Supplementary material for: Comprehensive analysis of CTNNB1 in adrenocortical carcinomas: Identification of novel mutations and correlation to survival
Source: Sci Rep. 2018 Jun 5;8:8610. doi: 10.1038/s41598-018-26799-2 (PMC5988720; doi:10.1038/s41598-018-26799-2)
Supplement: Supplementary file 1 — Supplementary Information [file 41598_2018_26799_MOESM1_ESM.pdf]

Supplementary Information

Comprehensive analysis of *CTNNB1* in adrenocortical carcinomas: Identification of novel mutations and correlation to survival.

Rajani Maharjan*, Samuel Backman, Tobias Åkerstöm, Per Hellman, Peyman Björklund.

Affiliations: Department of Surgical Sciences, Uppsala University, Uppsala, Sweden.

Corresponding Author:

Rajani Maharjan

Experimental Surgery Group, Department of Surgical Sciences.

Rudbeck Laboratory, R3

75185 Uppsala, Sweden.

Phone: +46184714706

Email: rajani.maharjan@surgsci.uu.se

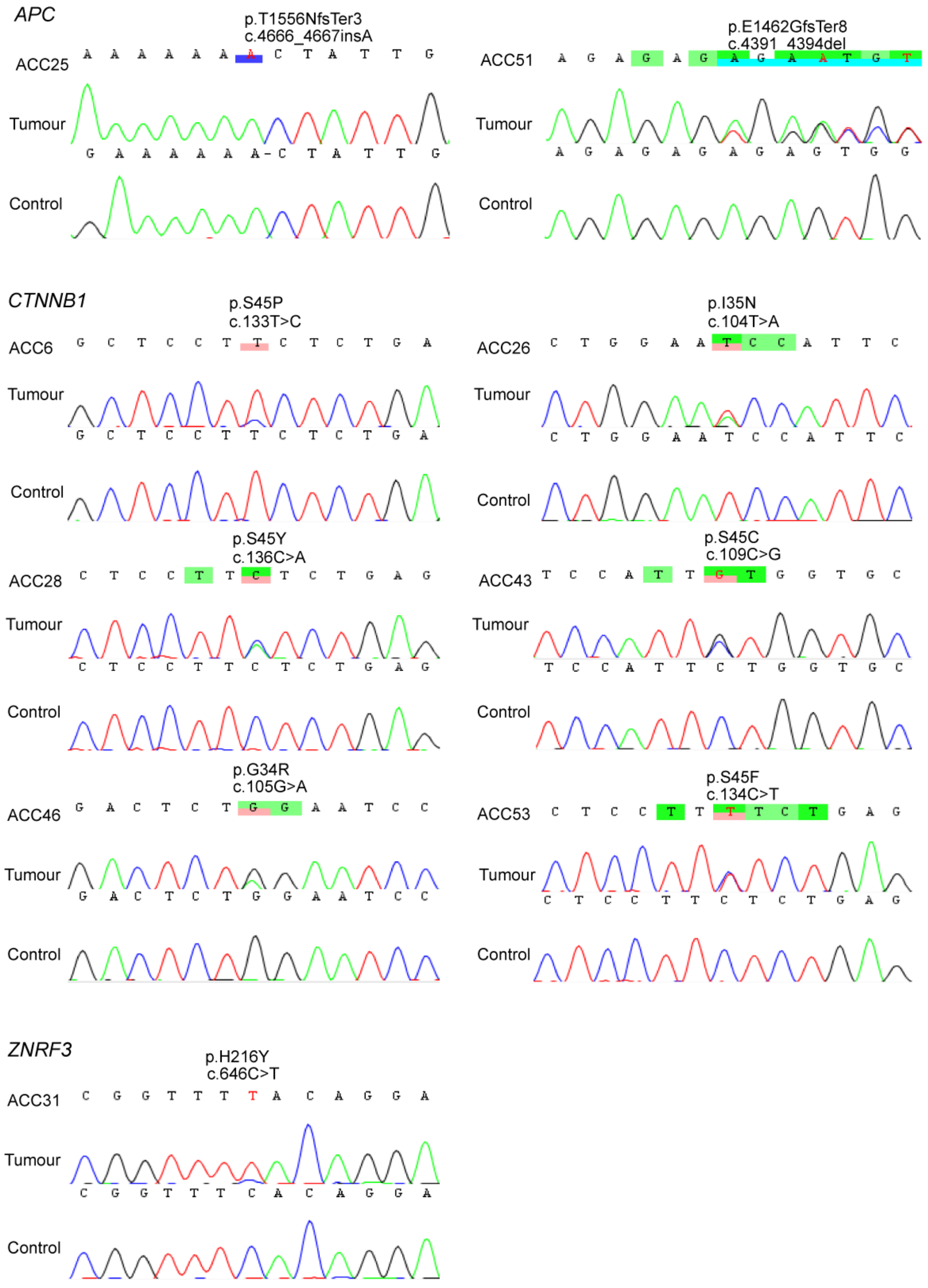

Supplementary Figure S1. Electropherogram showing *CTNNB1*, *ZNRF3* and *APC* mutations.

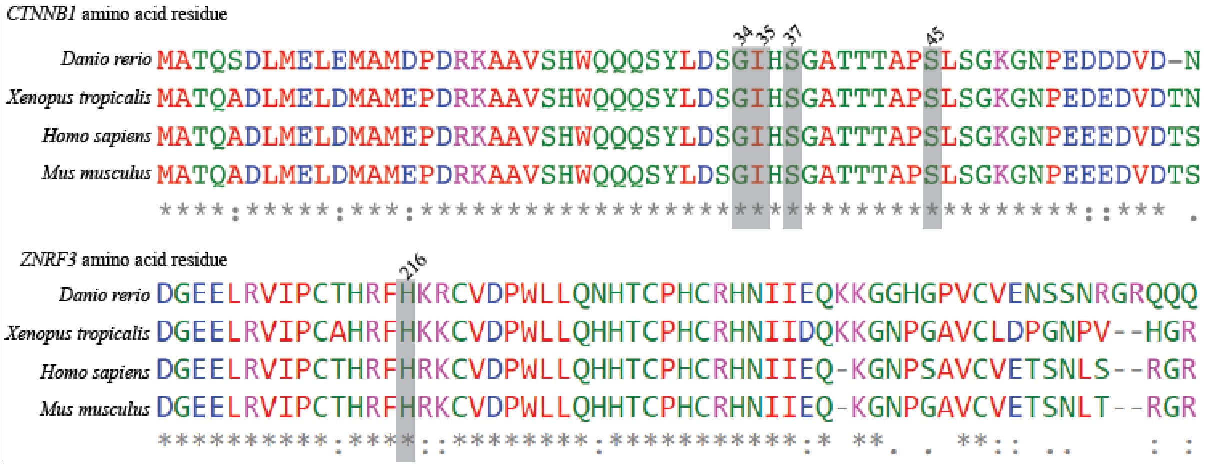

Supplementary Figure S2. Protein sequence alignment and comparison between *Danio rerio*, *Xenopus tropicalis*, *Homo Sapiens* and *Mus musculus*. The amino acid residues affected by the mutations are highlighted.

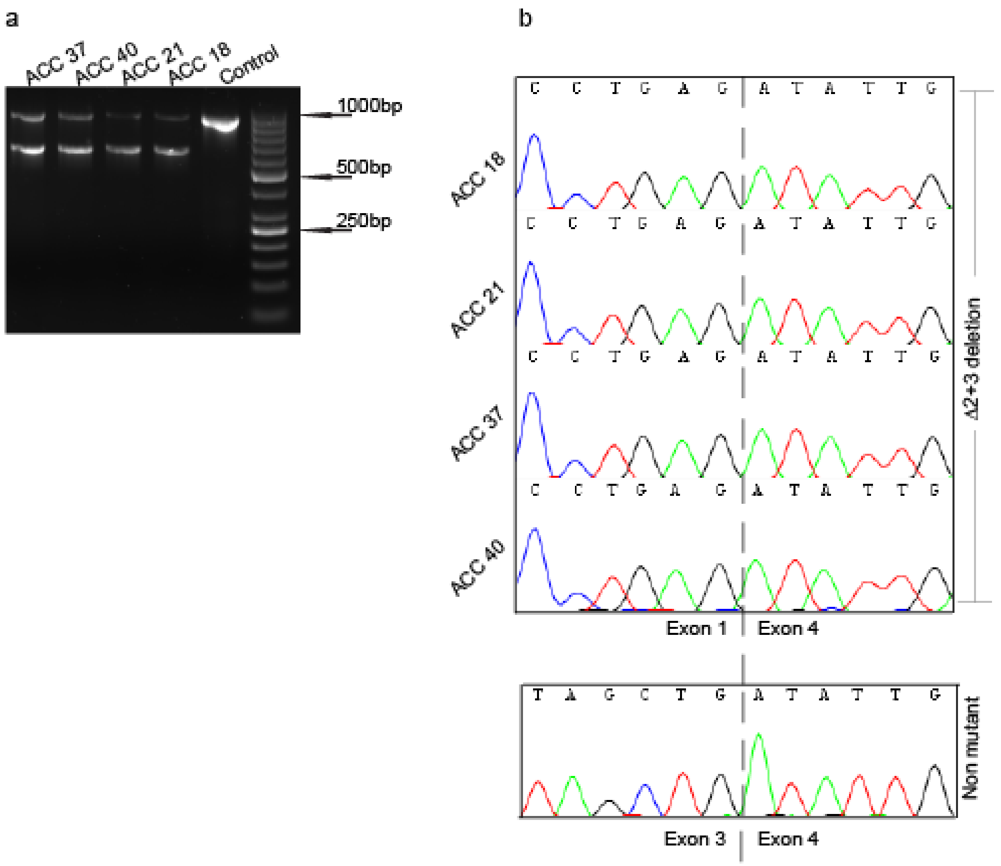

Supplementary Figure S3a) Gel electrophoresis on the cDNA. PCR products showing four ACC tumours with shorter product (630 bases) in addition to the wildtype product (920 bases). b) Electropherogram of ACC 18, ACC 21, ACC 37 and ACC 40 showing deletion of exon 2 and 3 in cDNA unlike non mutant control.

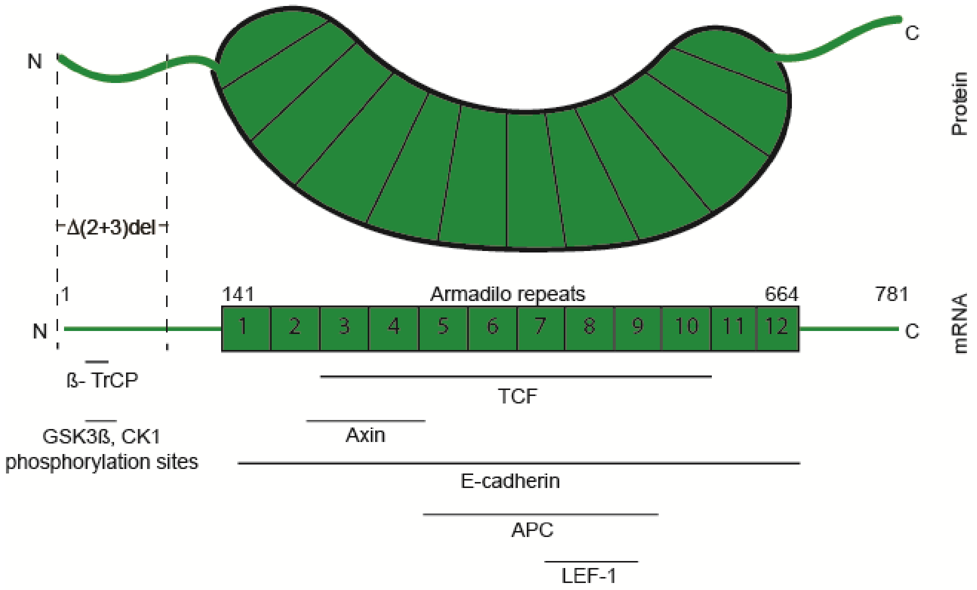

Supplementary Figure S4. Schematic illustration showing simplified β-Catenin mRNA and protein structure (based on crystal structure studies[1,2]) with different binding regions and Δ(2+3) deletion site.

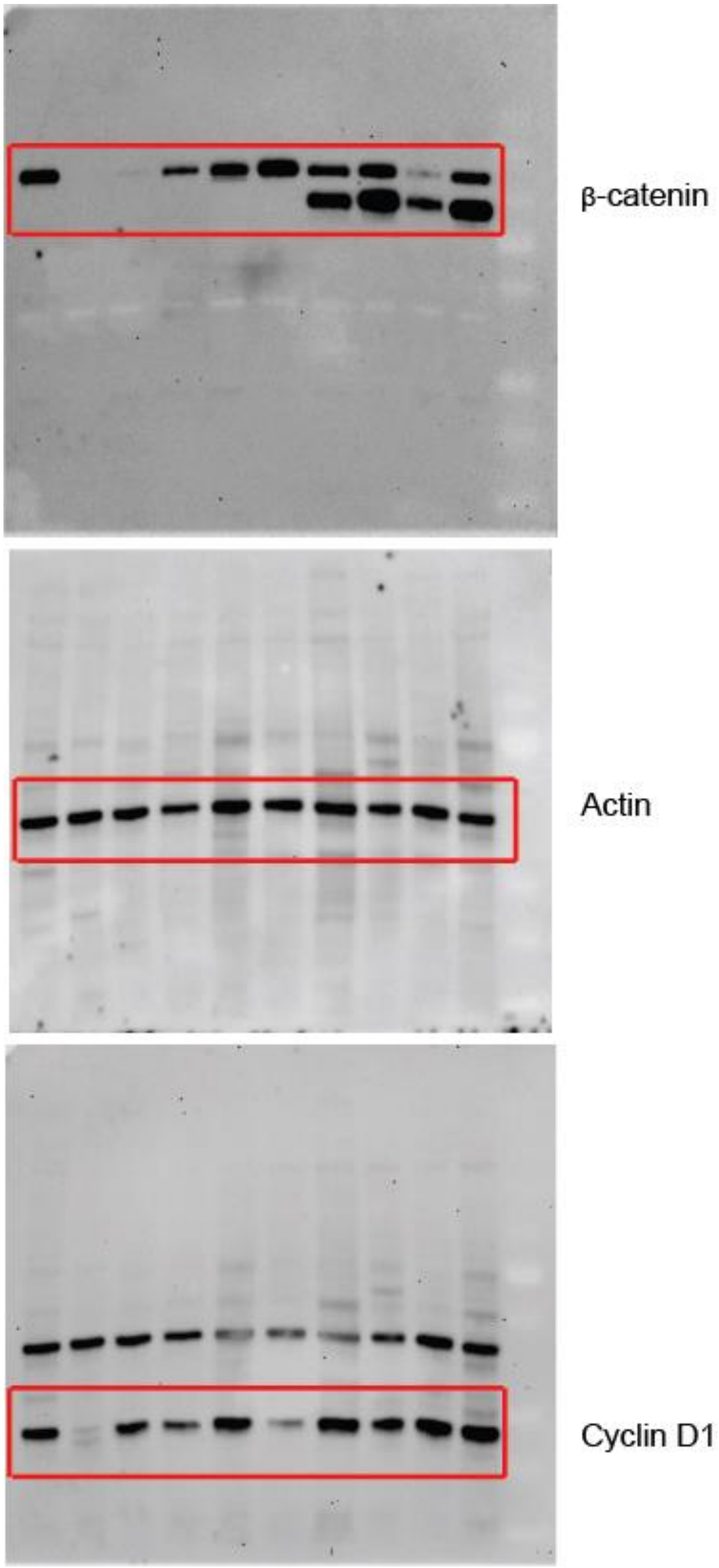

Supplementary Figure S5. Original Western blot for Figure 3a.

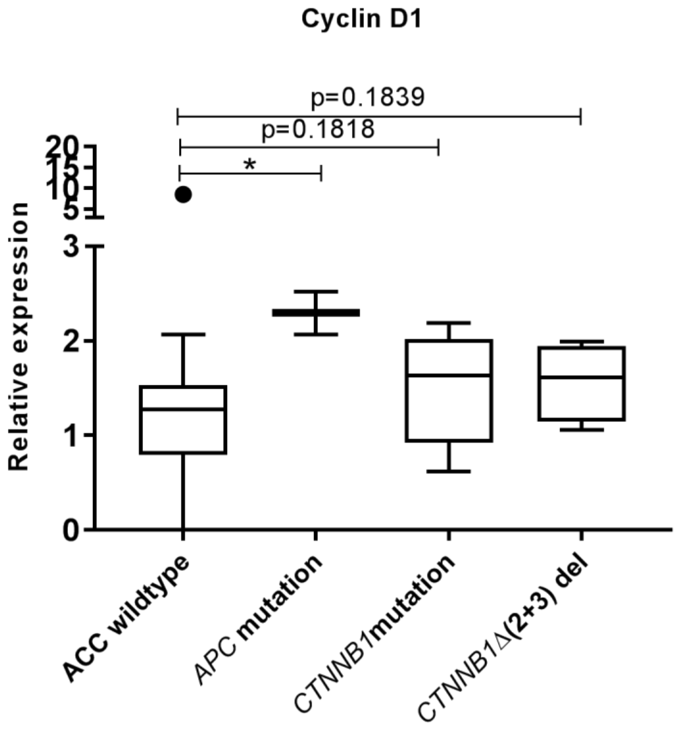

Supplementary Figure S6. mRNA expression of Cyclin D1 in tumour samples harboring *APC* mutation, *CTNNB1* mutation and Δ(2+3) deletion in comparison to rest of the cohort devoid of *CTNNB1* mutation or deletion (*, p=0.0144).

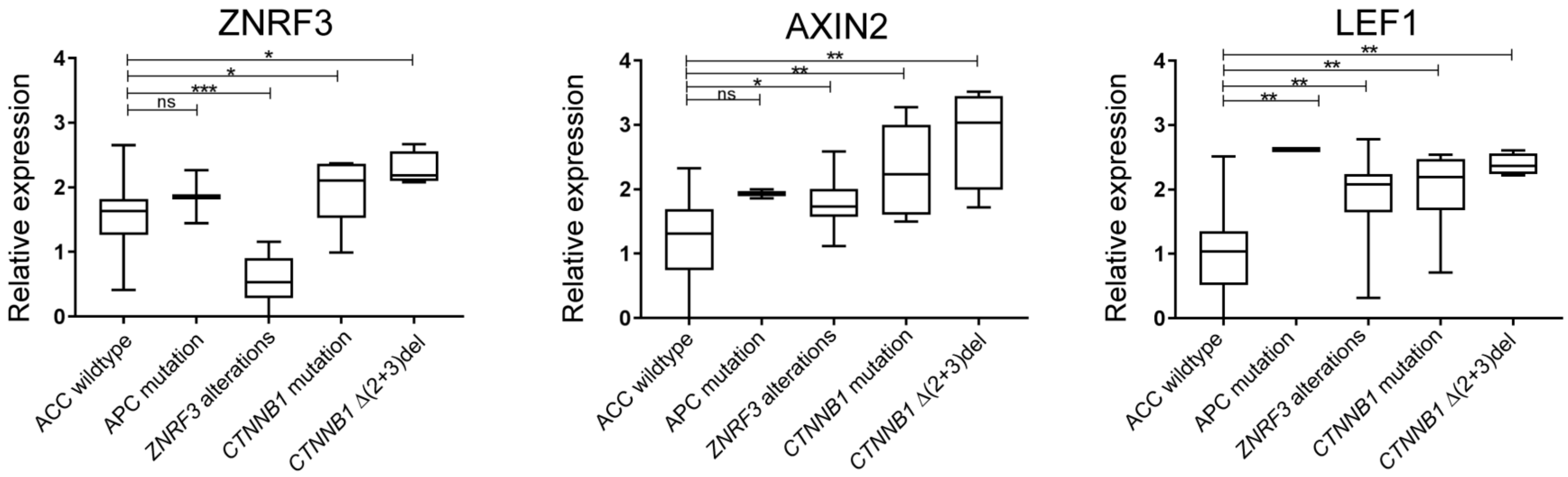

Supplementary Figure S7. mRNA expression of ZNRF3, AXIN2 and LEF1 in tumour samples harboring *APC* mutation, *ZNRF3* alterations, *CTNNB1* missense mutation and Δ(2+3) deletion in comparison to rest of the cohort (p values for AXIN2 and ZNRF3 for ACC wildtype vs *APC* mutation are 0.1239 and 0.5813 respectively).

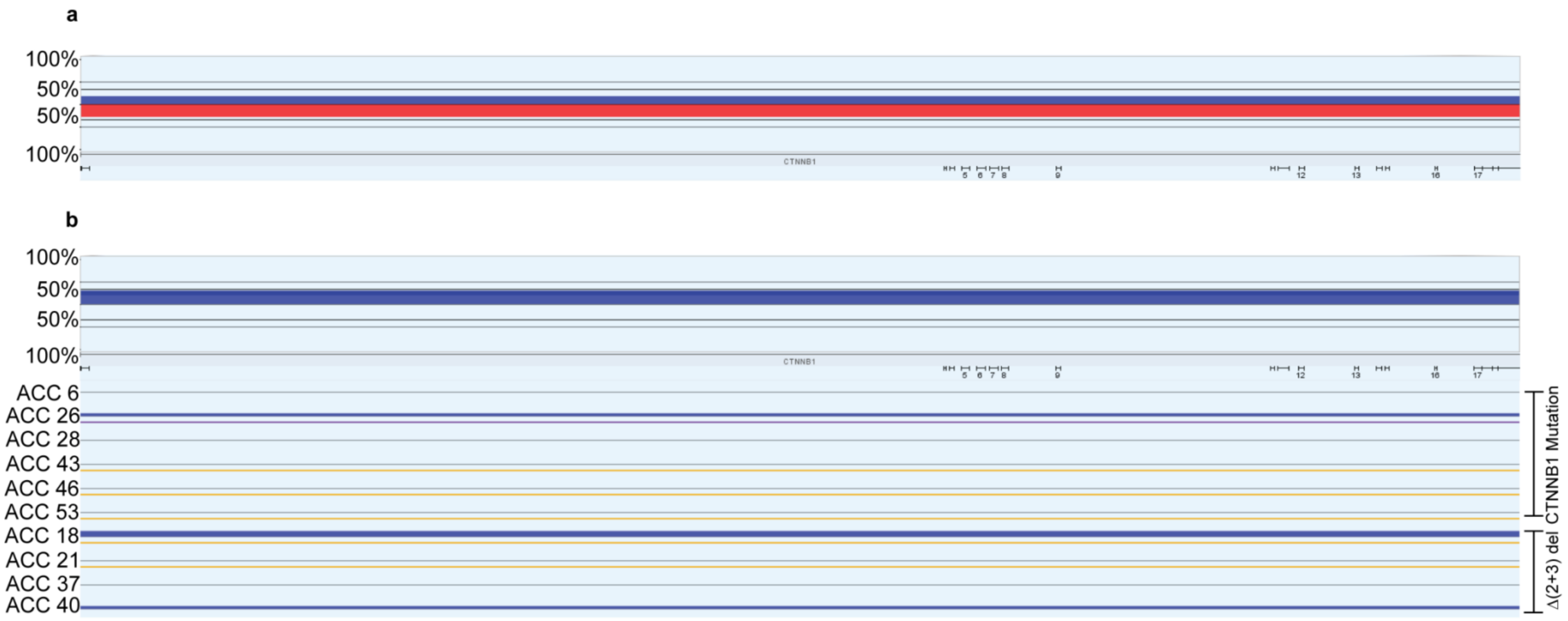

Supplementary Figure S8. SNP array analysis showing a) summary of events at *CTNNB1* locus 3p22.1 in the tumours without *CTNNB1* mutations b) summary of events at *CTNNB1* locus 3p22.1 for tumours with *CTNNB1* missense mutation and Δ(2+3) deletion (top panel) followed by details of each mutant sample. In the figure; red represents loss, blue represents gain and tan yellow represents loss of heterozygosity.

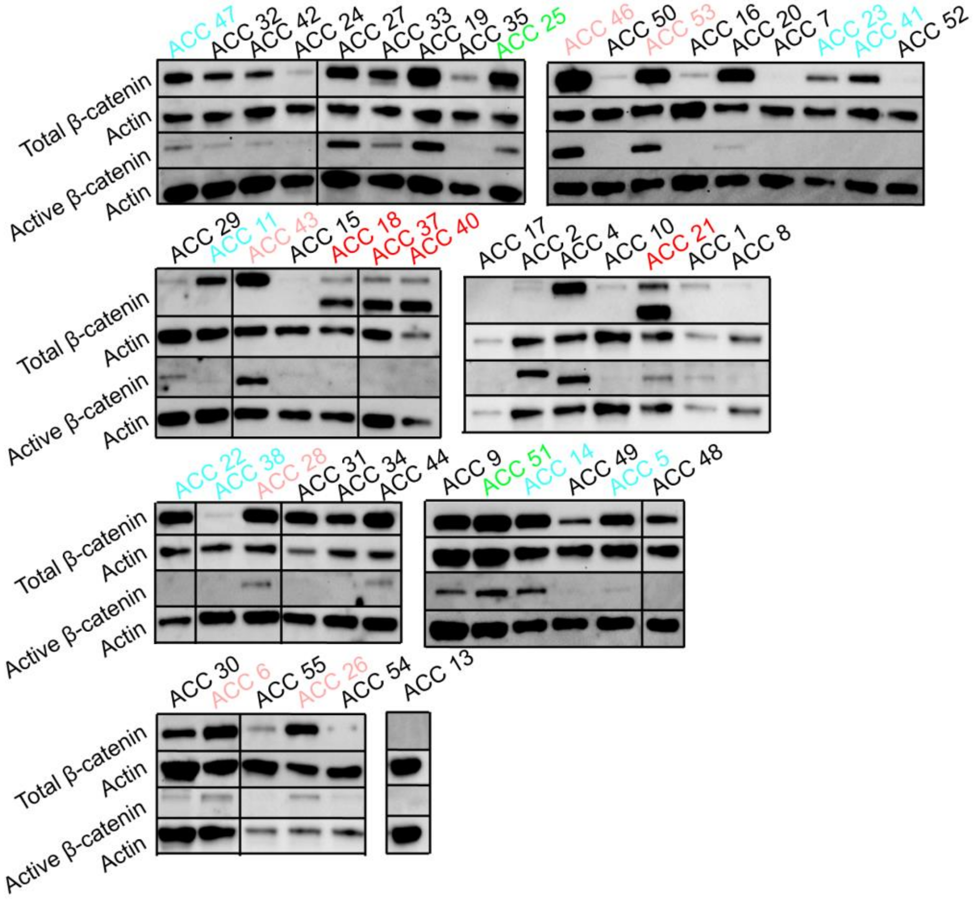

Supplementary Figure S9. Western blot on ACC tumours showing expression levels of total and active β-catenin and actin. The samples with APC mutation, *CTNNB1* missense mutation, *CTNNB1* Δ(2+3) deletion and *ZNRF3* deletions are highlighted in green, pink , red and blue respectively. Uncropped original blots are shown in Supplementary Fig S16.

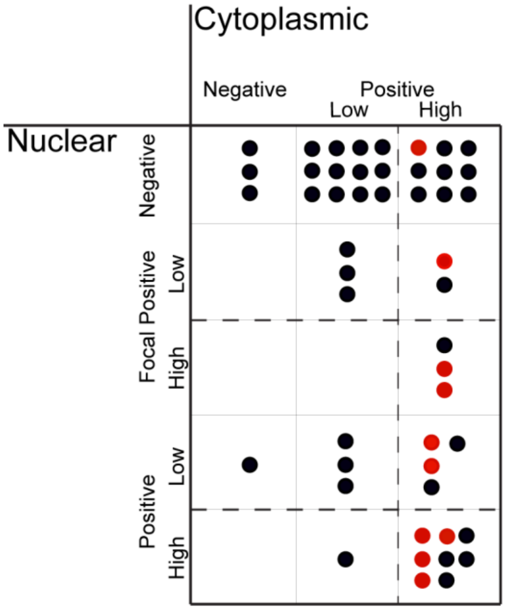

Supplementary Figure S10. Tumours grouped according to the immunohistochemical staining of β-catenin. *CTNNB1* mutants are marked in red and the rest of the samples are marked in black. The samples were scored and categorized as “negative” or “positive” for cytoplasmic staining and as “negative”, “focal positive” (heterogeneous) and “positive” for nuclear staining. For positive cases the nuclear and cytoplasmic staining intensity were scored manually ascending from 1+ to 5+. Samples with 1+ to 2+ scoring were grouped as “low” and those with 3+to 5+ scoring were grouped as “high”.

a)

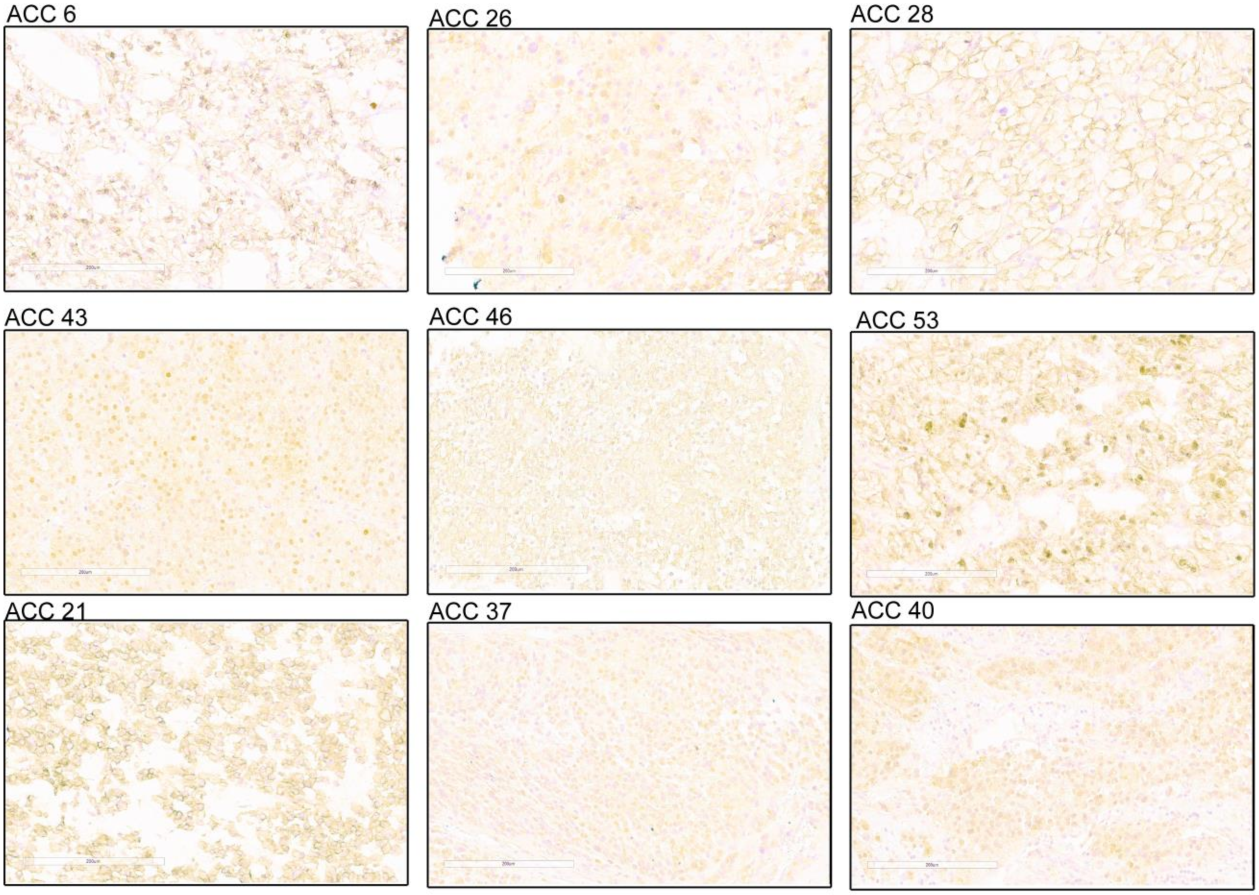

b)

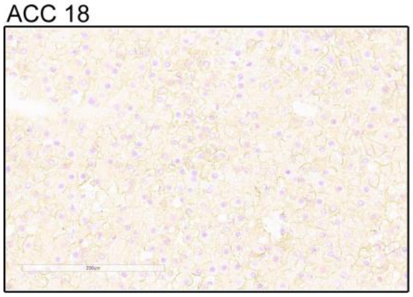

c)

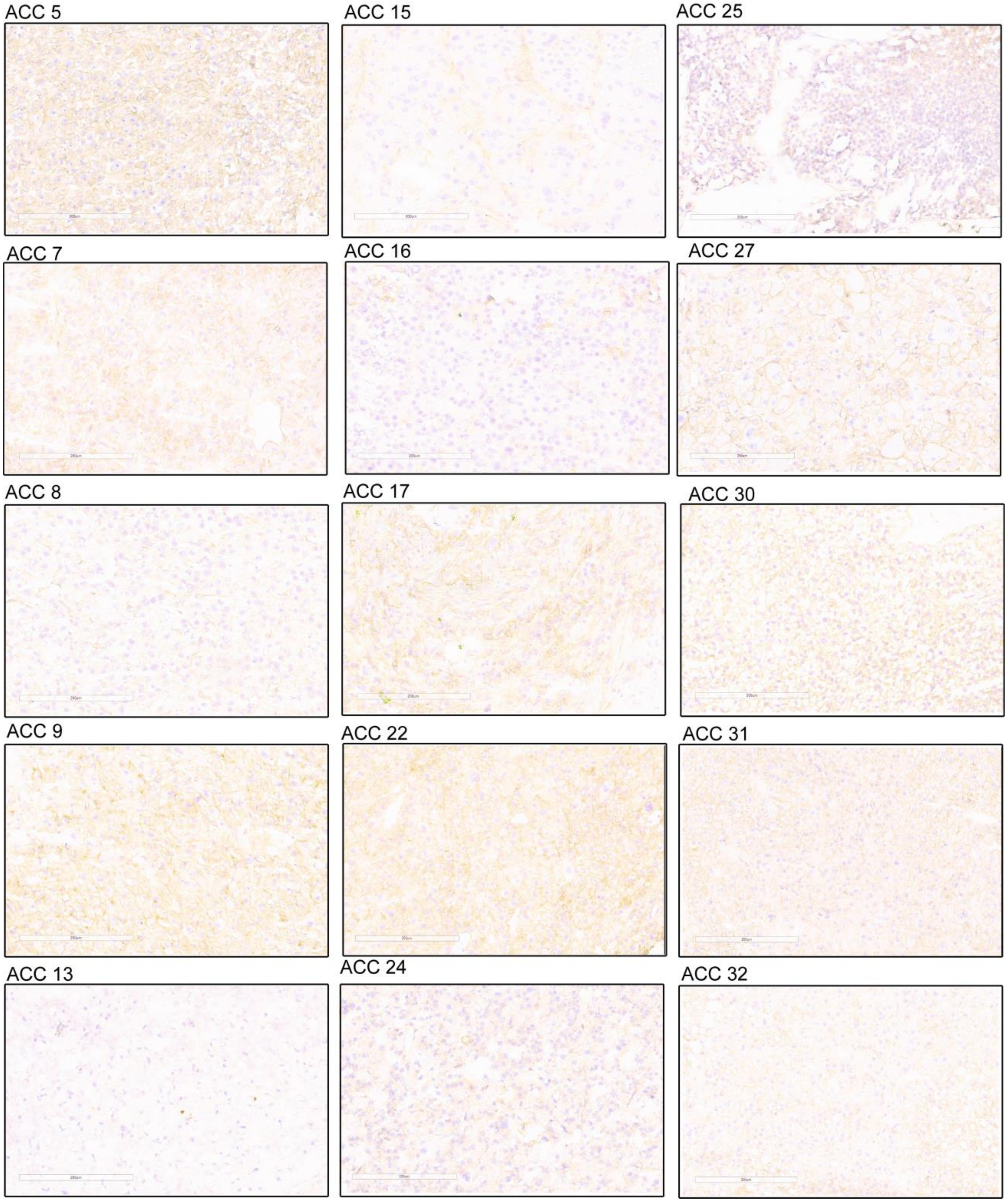
ACC 5
ACC 15
ACC 25
ACC 7
ACC 16
ACC 27
ACC 8
ACC 17
ACC 30
ACC 9
ACC 22
ACC 31
ACC 13
ACC 24
ACC 32

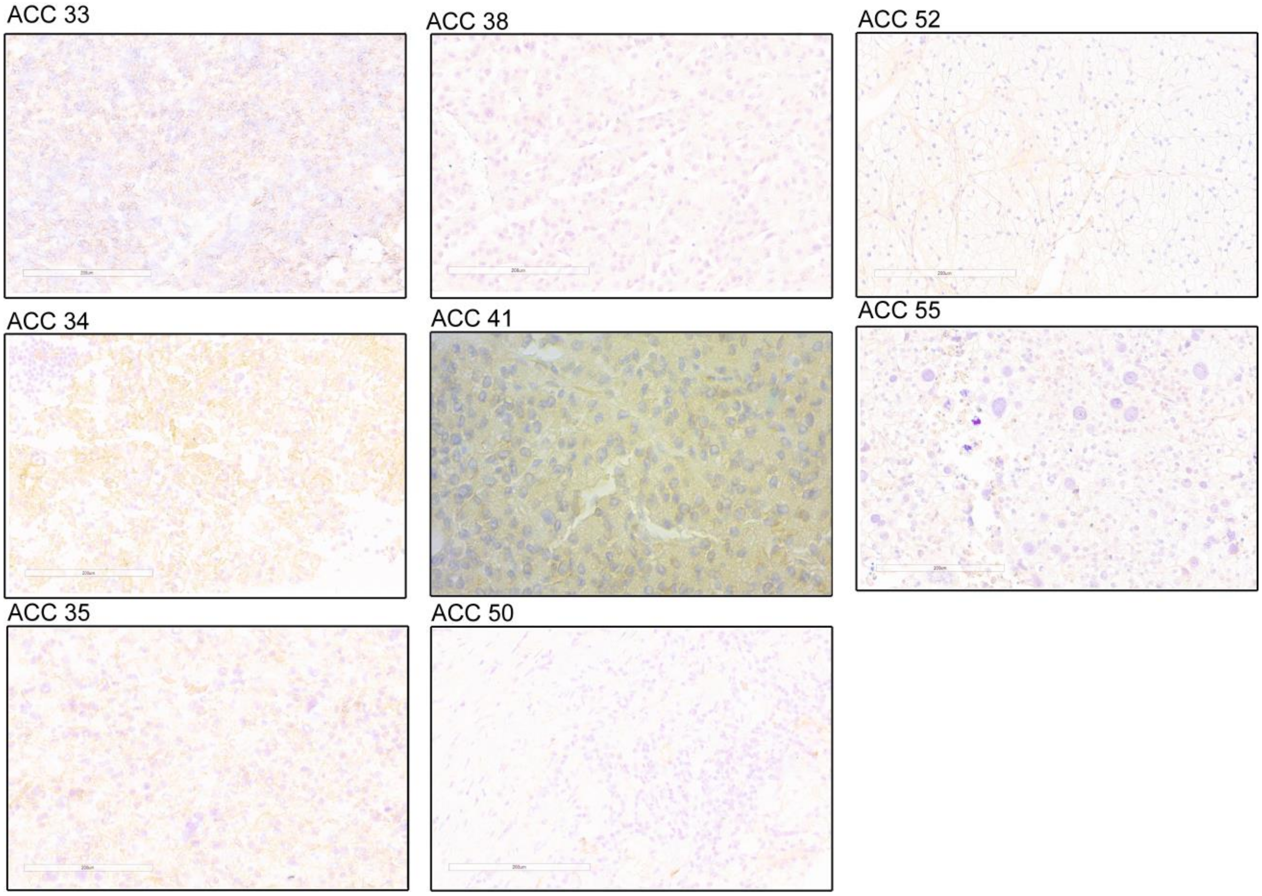
ACC 33
ACC 38
ACC 52
ACC 34
ACC 41
ACC 55
ACC 35
ACC 50

d)

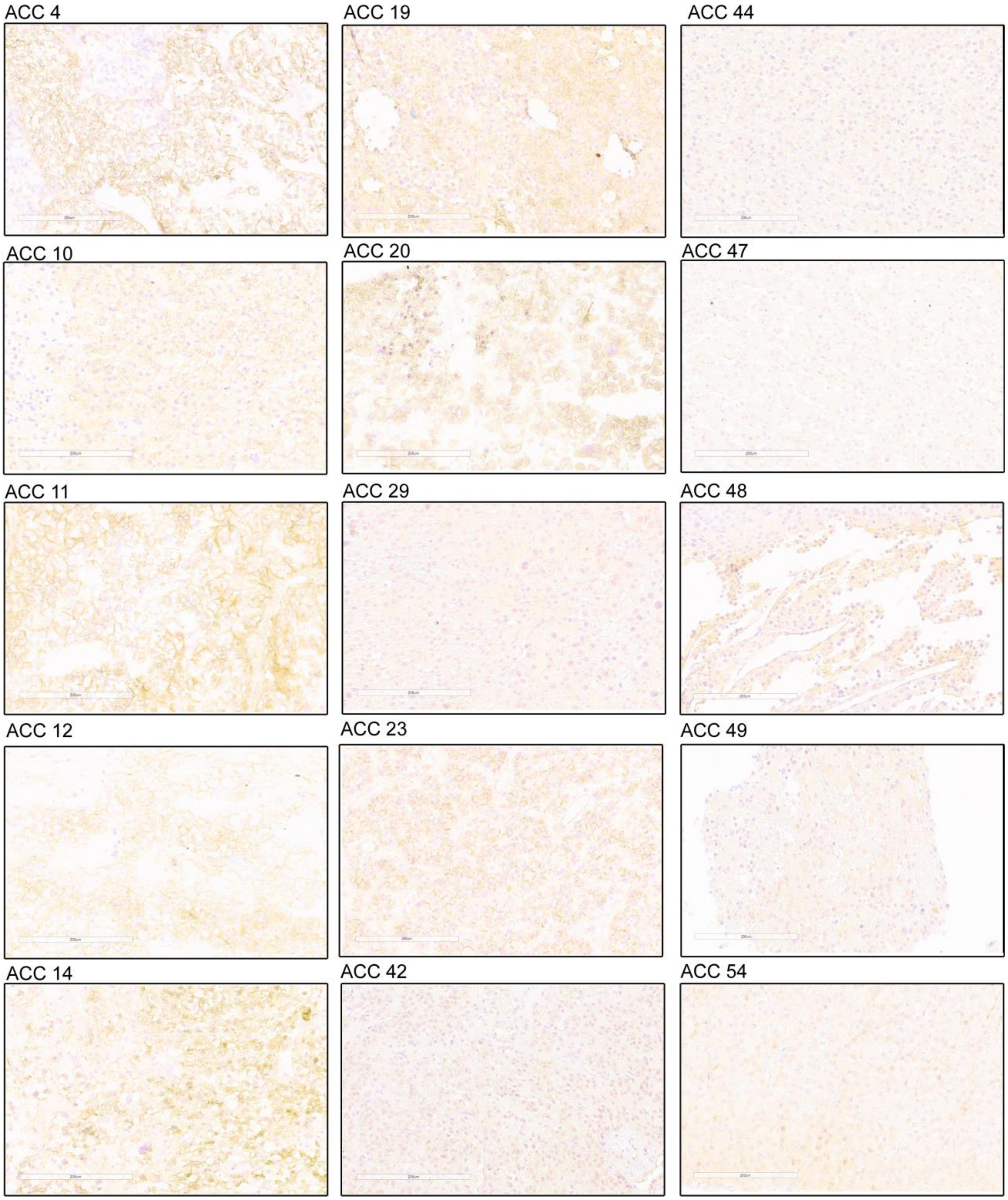
ACC 4
ACC 19
ACC 44
ACC 10
ACC 20
ACC 47
ACC 11
ACC 29
ACC 48
ACC 12
ACC 23
ACC 49
ACC 14
ACC 42
ACC 54

ACC 51

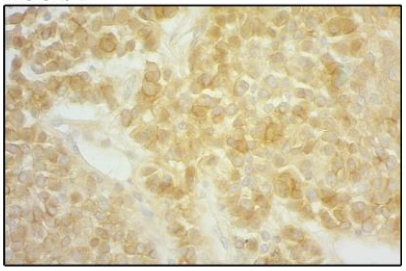

Supplementary Figure S11. Photomicrographs showing different groups of ACC in terms of β-catenin nuclear expression. a) tumours with *CTNNB1* mutation and nuclear expression b) tumour with *CTNNB1* mutation but without nuclear expression; c) tumours without *CTNNB1* mutation and nuclear expression d) tumours without *CTNNB1* mutation and with nuclear expression.

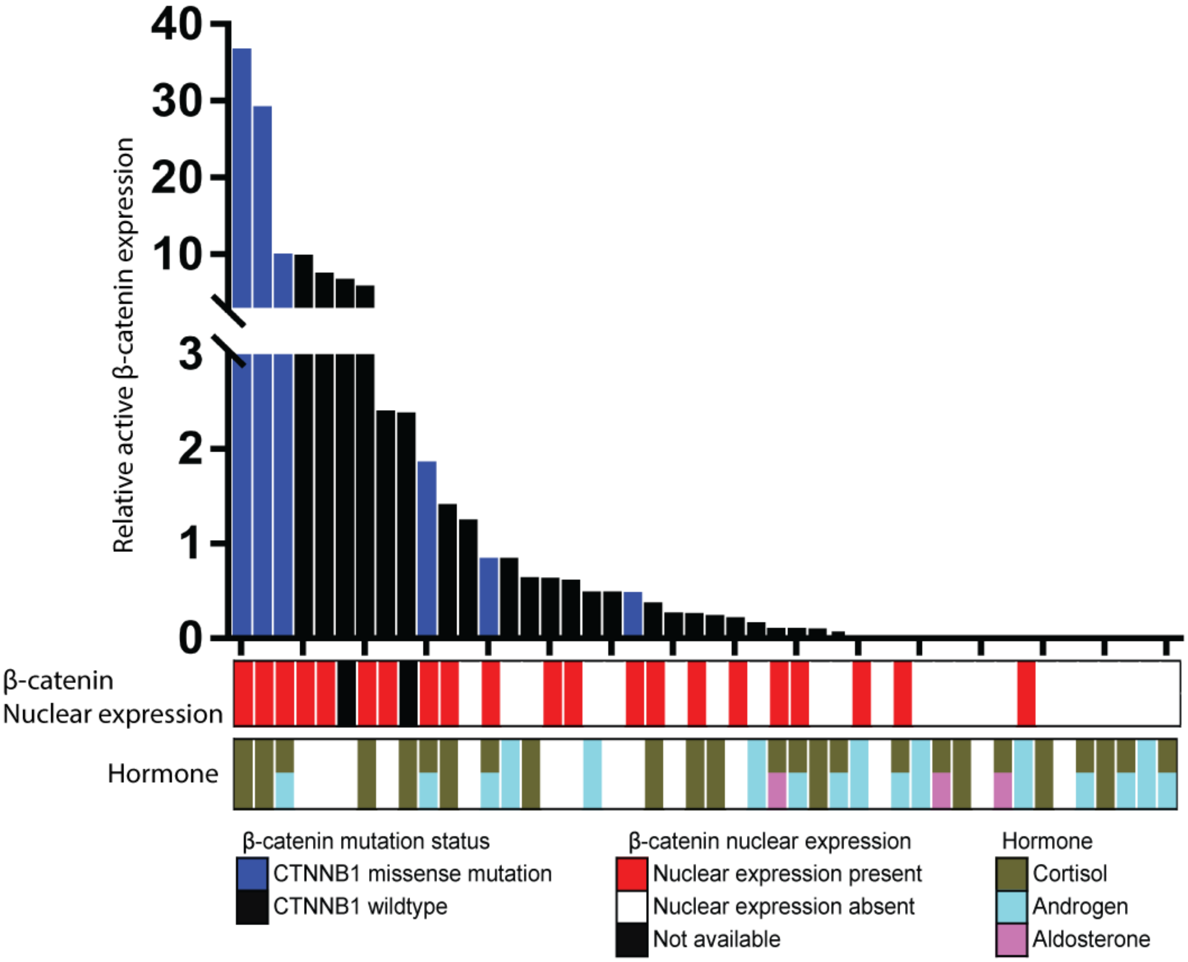

Supplementary Fig S12: Comparative analysis of active beta catenin expression and nuclear expression in ACC.

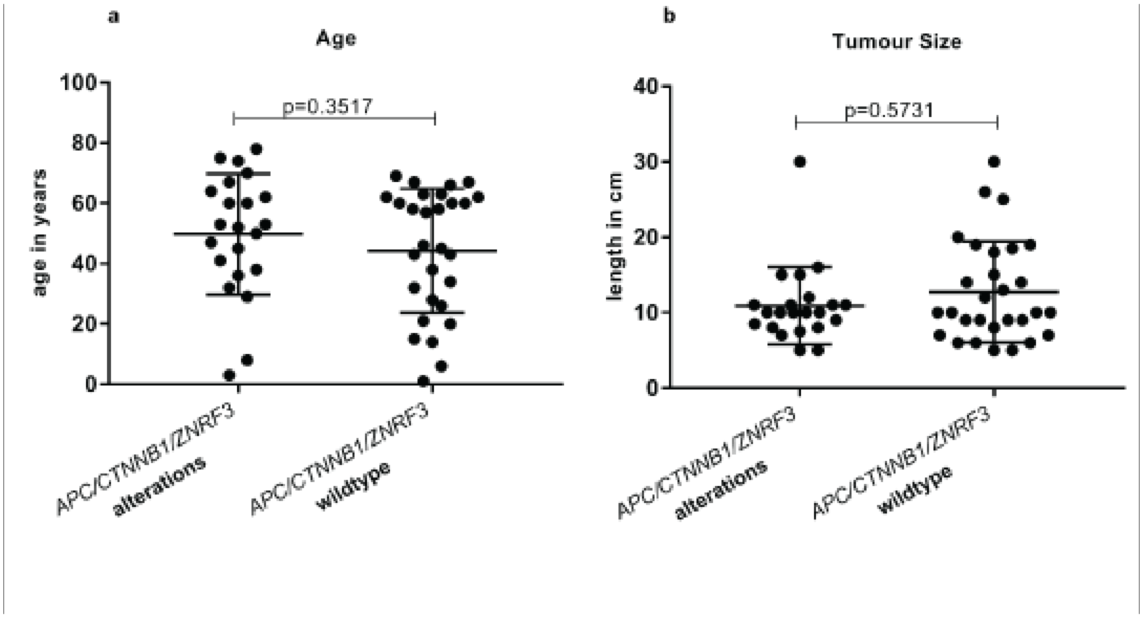

Supplementary Figure S13. a) Age at operation and b) tumour size comparison between group of tumours with *APC*/*CTNNB1/ZNRF3* alterations versus tumours wildtype for *APC*/*CTNNB1/ZNRF3*.

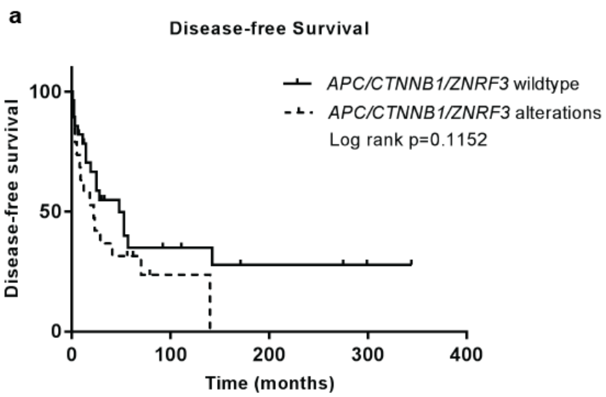

Supplementary Figure S14. Disease-free survival graph for a) patients with tumour harbouring *APC/CTNNB1/ZNRF3* alterations versus the wildtype b) patients with tumour harbouring *CTNNB1* mutations alone versus the wildtype.

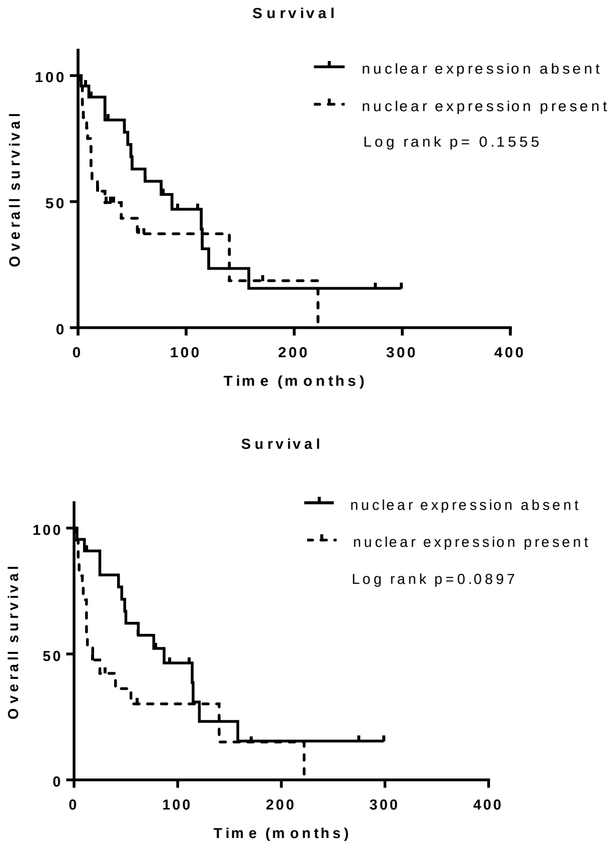

Supplementary Figure S15. Overall survival graph for patients with tumour showing nuclear expression of β-catenin versus those lacking the expression in whole cohort (top) and in adult ACC (bottom).

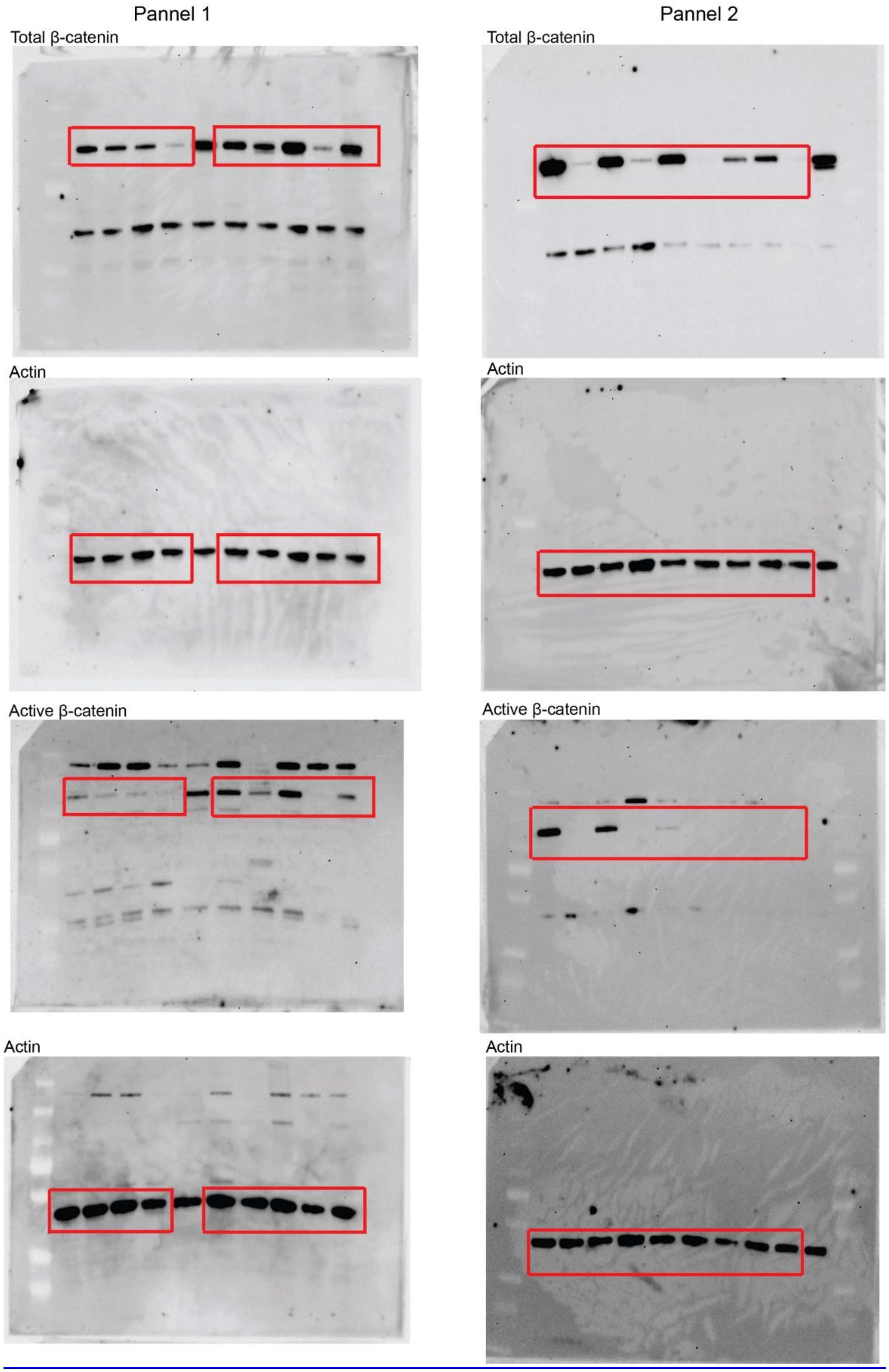
Pannel 1
Pannel 2
Total β-catenin
Total β-catenin
Actin
Actin
Active β-catenin
Active β-catenin
Actin
Actin

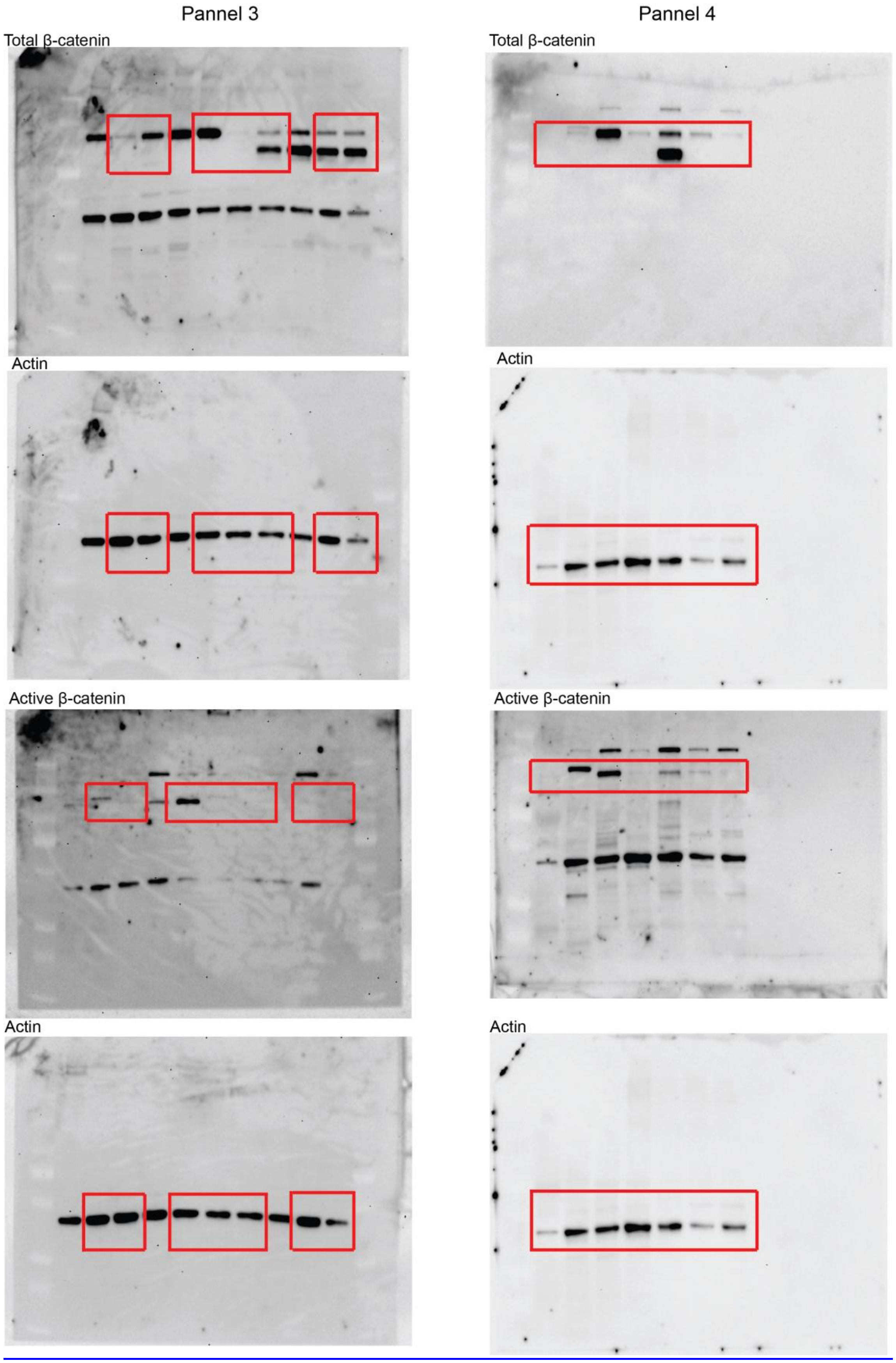

Pannel 3
Pannel 4
Total β-catenin
Total β-catenin
Actin
Actin
Active β-catenin
Active β-catenin
Actin
Actin

Pannel 5

Total β-catenin

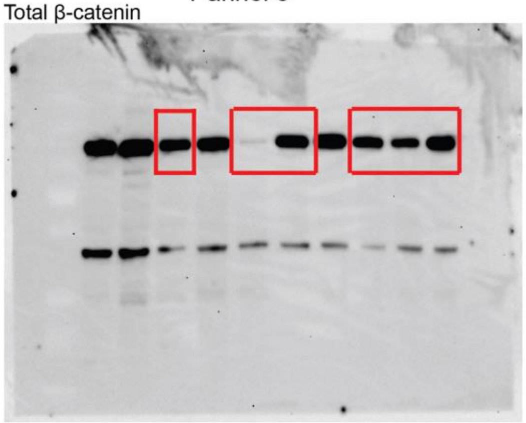

Actin

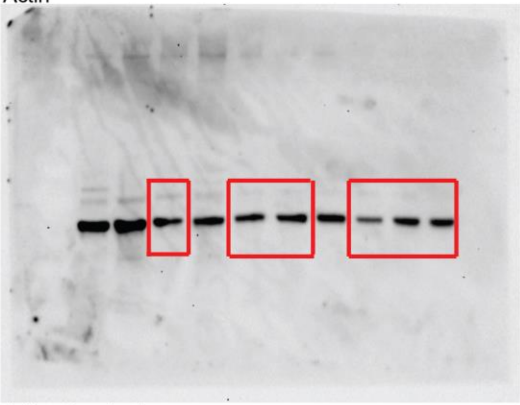

Active β-catenin

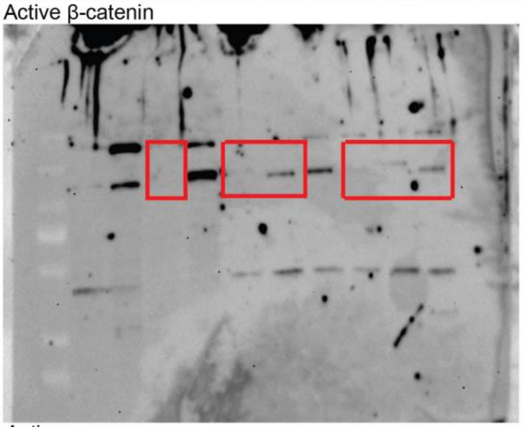

Actin

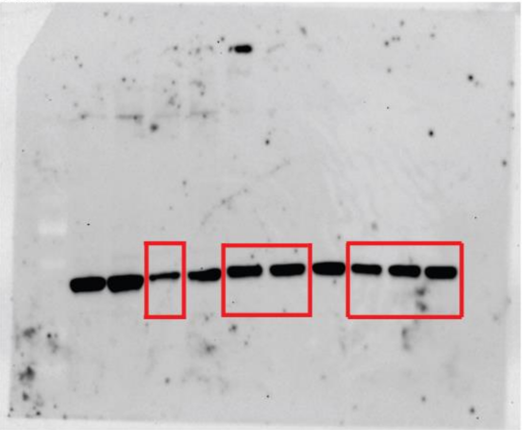

Pannel 6

Total β-catenin

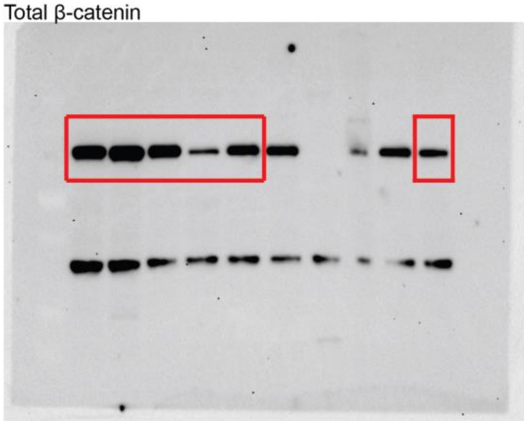

Actin

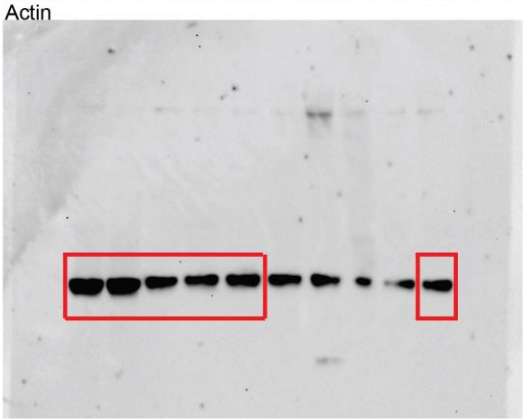

Active β-catenin

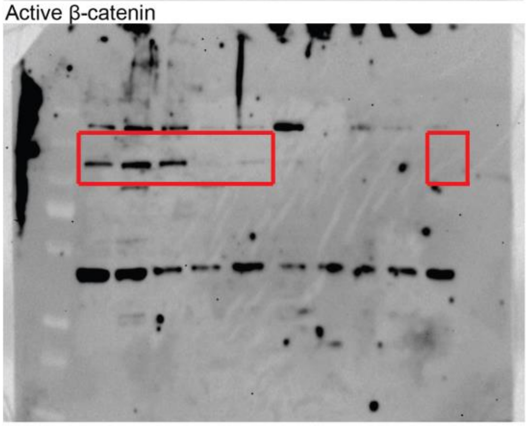

Actin

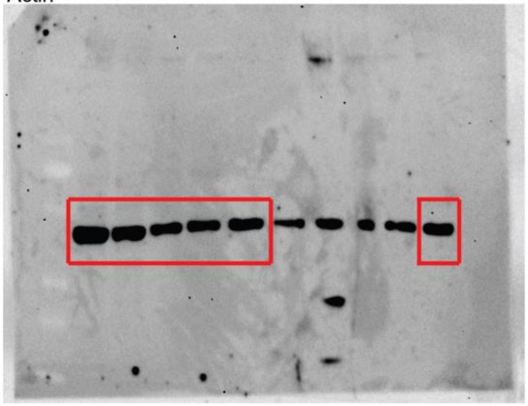

Pannel 7

Total β-catenin

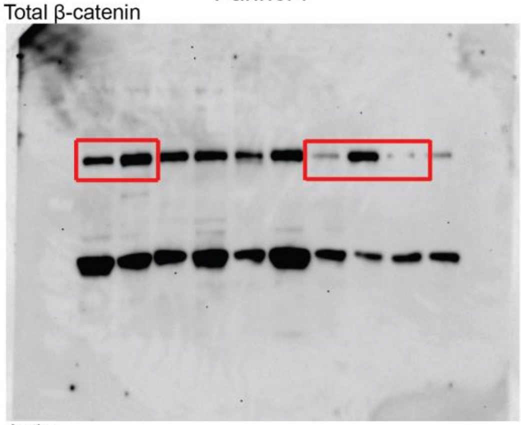

Actin

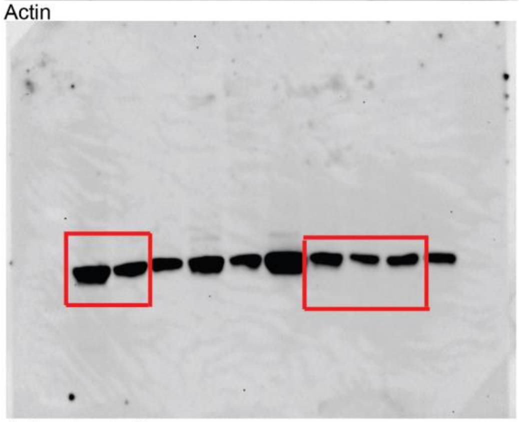

Active β-catenin

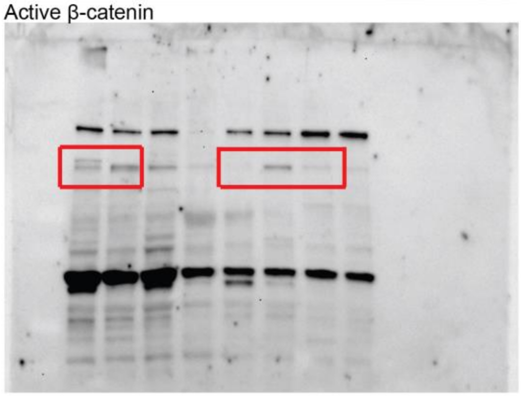

Actin

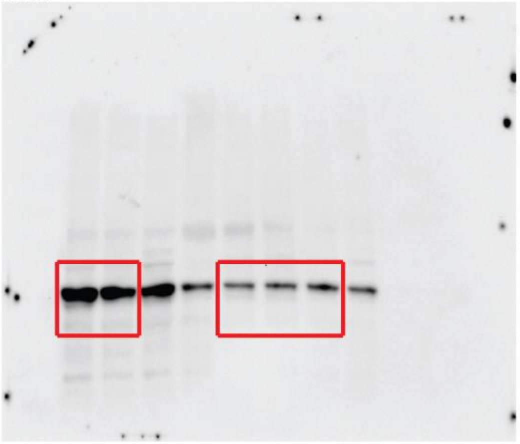

Pannel 8

Total β-catenin

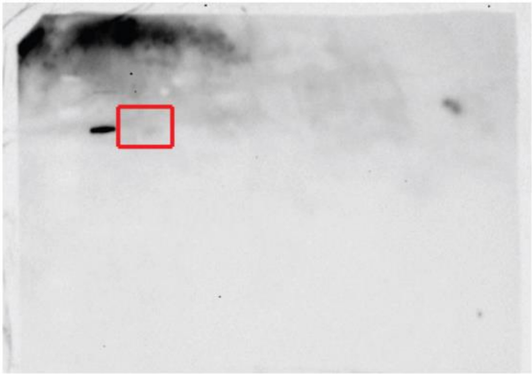

Actin

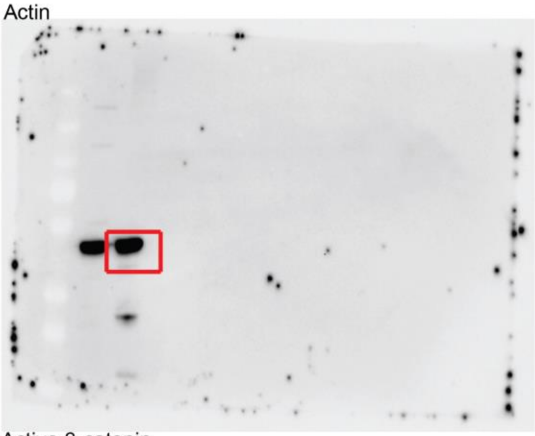

Active β-catenin

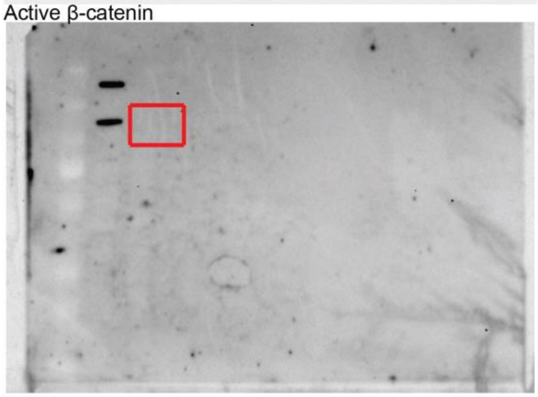

Actin

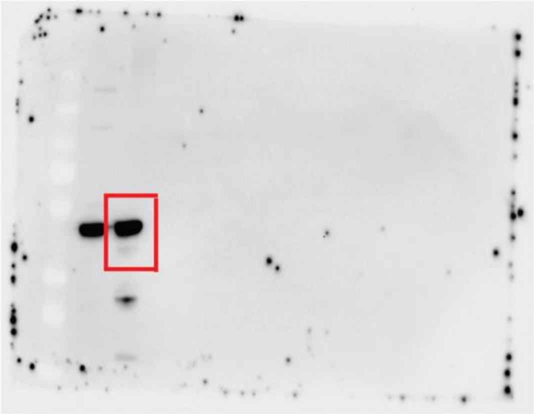

Supplementary Figure S16. Original blots for Supplementary Figure S9. The samples enclosed by the red box represent the samples included in Supplementary Figure S9. Panel 1, 2, 3, 4, 5, 6, 7, and 8 in the figure corresponds to the panel comprising samples ACC 47 - ACC 25, ACC 46 - ACC 52, ACC 29 – ACC 40, ACC 17- ACC 8, ACC 22 - ACC 44, ACC 9 - ACC 48, ACC 30 – ACC 54, and ACC 13 in Supplementary Figure S9 respectively.

Supplementary Table S1: Missense mutation details

Sample ID	*CTNNB1* mutation		Mutation Type	SIFT	Polyphen-2	PROVEAN
	**cDNA**	**Protein**				
ACC6	c.133T>C	p.S45P	Missense	Damaging (0)	Prob. Damaging (0.988)	Deleterious (-2.928)
ACC 26	c.104T>A	p.I35N	Missense	Damaging (0)	Prob. Damaging (1.00)	Deleterious (-5.175))
ACC 28	c.136C>A	p.S45Y	Missense	Damaging (0)	Prob. Damaging (0.999)	Deleterious (-3.620)
ACC 43	c.109C>G	p.S37C	Missense	Damaging (0)	Prob. Damaging (1.00)	Deleterious (-3.951)
ACC 46	c.105G>A	p.G34R	Missense	Damaging (0)	Prob. Damaging (1.00)	Deleterious (-6.200)
ACC 53	c.134C>T	p.S45F	Missense	Damaging (0)	Prob. Damaging (0.996)	Deleterious (-3.849)
**Sample ID**	***ZNRF3* mutation**		**Mutation Type**	**SIFT**	**Polyphen-2**	**PROVEAN**
	**cDNA**	**Protein**				
ACC31	c.646C>T	p.H216Y	Missense	Damaging (0)	Prob.Damaging (0.999)	Deleterious (-5.577)
**Sample ID**	***APC* mutation**		**Mutation Type**	**SIFT**	**Polyphen-2**	**PROVEAN**
	**cDNA**	**Protein**				
ACC25	c.4666_4667insA	p.T1556NfsTer3	Insertion	-	-	-
ACC51	c.4391_4394del	p.E1462GfsTer8	Deletion	-		

**Supplementary Table S2: Primer used for detection of deletion in DNA**	
ACC 18	
Fw primer	GCTTCACTGTATGTATGATC
Rw primer	CTGACTTTCAGTAAGGCAATG
ACC 21	
Fw primer	CTAGAACTTCTGGTGATATGG
Rw primer	CTGTGTAGATGGGATCTGC
ACC 37	
Fw primer	CTGCTAGTTTCACCATATCC
Rw primer	CTCACTATCCACAGTTCAGC
ACC 40	
Fw primer	CTGCTAGTTTCACCATATCC
Rw primer	CTGACTTTCAGTAAGGCAATG

**Supplementary Table S3: Details of deletion mutation**			
Sample	Deletion (genome)	Mutation(coding DNA)	Mutation(RNA)
ACC18	g.41217632_g.41224675del	c.-48-6389_163del	r.-48_241del
ACC21	g. 41220754_g.41224725delinsA	c.-48-3267_213del	r.-48_241del
ACC37	g. 41218557_g. 41224902delinsT	c.-48-5464_241-51del	r.-48_241del
ACC40	g.41218317_g.41224723del	c.-48-5704_211del	r.-48_241del
Transcript ID: ENST00000349496.9			

Supplementary Table S4. Analysis of sequences at deletion junctions.		
Sample	Deletion	Sequence at the site of deletion
ACC18	g.41217632_g.41224675del	CAAAGGGTAGttacacttta……..cctgaggaagAGGATGTGG
ACC40	g.41218317_g.41224723del	TAATACAATActtgaattgc……..cagggattttCTCAGTCCTT
ACC37	g. 41218557_g.41224902delinsT	GTTAAA**CTAAg**agttgtttt……..tagcaaata**cTTAG**GTAAA
ACC21	g.41220754_g.41224725delinsA	ATTATGAAT**Tg**ctagtataa……..gggattttct**CA**GTCCTTCA

Two of the samples harboring the Δ(2+3) deletion (ACC 18, ACC 40) displayed sequence microhomology whereas the other two samples (ACC 37, ACC 21) harbored reverse complement sequence at the breakpoint. The sequences in lowercase font depict the deleted regions and the uppercase font on the left and right depict deletions start and end sites. The microhomologous sequences are underlined whereas the reverse complement sequences are bold and underlined. The samples harboring reverse complement sequences also had similar patterns of deletion where an additional nucleotide was inserted at the breakpoint, unlike the ones displaying microhomology that harbored a defined breakpoint.

Supplementary Table S5: Sequence at deletion site.	
ACC18	
	deletion start
CAAAGGGTAG	TTACACTTTA
CCTGAGGAAG	AGGATGTGGA
deletion end	
ACC21	
	deletion start
ATTATGAATT	GCTAGTATAAAA
GGGATTTTCT	CAGTCCTTCACT
deletion end	
ACC37	
	deletion start
GTTAAACTAA	GAGTTGTTTT
TAGCAAATAC	TTAGGTAAAT
deletion end	
ACC40	
	deletion start
TAATACAATA	CTTGAATTGC
CAGGGATTTT	CTCAGTCCTT
deletion end	

Several small identical (flanking microhomology), complement or reverse complement sequences near the breakpoints were observed. Similar sequence are highlighted in red, reverse complement sequence in green and complementary sequence in blue

Supplementary Table S6: Nucleotide percentile in Deletion start site					
Tumours	Nos. (T)	Nos. (A)	Nos. (G)	Nos. (C)	AT(percent)
ACC18	36T	34A	17G	13C	70%
ACC21	43T	30A	20G	07C	73%
ACC 37	31T	35A	21G	13C	72%
ACC 40	47T	25A	11G	17C	72%

Supplementary Table S7. Cohort details

ACC case	Mutations	Gene	Nuclear Accumulation	Age	Gender	Event (OS) (yr/mnth)	DFS (yr/mnth)	Tumour Size(cm)	TNM	ENSAT Stage	Hormone Production
1	No		NA	32	M	28 y/8 m	28 y/8 m	7x5x5	T2 N0 M0	II	Cortisol
2	No		NA	60	F	Ɨ 11 y/10 m	†11y10m	9x8x6	T2 N0 M0	II	None
3	No		NA	45	F	Ɨ 1 y/2 m	†1y/2 m	10x6x3	T2 N0 M0	III	Cortisol
4	No		+	66	F	Ɨ 18 y/6 m	†4y/5m	7x6	T2 N0 M0	II	None
5	No		-	53	F	Ɨ 7 y/3 m	†2y/5m	4.5x7.5x10	T3 N0 M0	III	Androgen
6	c.133T>C	*CTNNB1*	+	32	F	Ɨ 8 m	†8m	8x6	T2 N0 M0	II	None
7	No		-	60	M	Ɨ 5 y/2 m	†4y	20x20	T2 N0 M0	II	Cortisol+Aldosteron
8	No		-	21	M	Ɨ 3 y/10 m	†6m	18.5x14x10	T2 N0 M0	II	Androgen
9	No		-	20	F	24 y/11 m	24y/11 m	5x5	T1 N0 M0	I	Androgen
10	No		+	69	F	Ɨ 1 y	†11m	30x25x20	T3 N1 M0	III	None
11	No		+	45	F	Ɨ 4 y/7 m	†3y/5m	9x5x4	T2 N0 M0	II	Cortisol+Androgen
12	No		+	60	F	Ɨ 3 y/4 m	†1y	9x9x10	T3N1M0	III	Androgen
13	No		-	63	F	Ɨ 2 y/3m	†2y/3m	6.5x5x9	T3N1M0	III	Cortisol
14	No		+	36	F	Ɨ 1 y/6 m	†1y/6 m	8.5x7x11	T3 N1 M0	III	None
15	No		-	46	M	Ɨ 4 y/2 m	†2y/4m	12x14	T3 N1 M0	III	Cortisol
16	No		-	58	F	22 y/8 m	22 y/8 m	5x5.5x6	T2 N0 M0	II	None
17	No		-	38	F	Ɨ 2 y/1 m	†2 y/1 m	10 x 5	T3 N0 M0	III	None
18	c.-48-6389_163del	*CTNNB1*	-	29	F	Ɨ 3 y/7 m	†9m	11x10x7	T2 N0 M0	II	Cortisol+Androgen
19	No		+	NA	F	NA	NA	NA	NA	NA	NA
20	No		+	62	M	Ɨ 2 y/1 m	†1y/2m	6x4x5	T3 N0 M0	III	Cortisol
21	c.-48-3267_213del	*CTNNB1*	+	41	M	Ɨ 1y	†5m	15x11x8	T3 N0 M0	III	Cortisol
22	No		-	52	M	Ɨ 4 y/1 m	†1y/10m	5x3.5	T3 N0 M0	III	Cortisol+Aldosterone
23	No		+	70	M	Ɨ 5 m	-	10x7x5	T4 N0 M1	IV	Androgen
24	No		-	26	F	Ɨ 10 y/1 m	-	10x14	T4 N0 M1	IV	Cortisol+Androgen

25	c.4666_4667insA	*APC*	-	47	F	Ɨ 6y/5 m	†5y/10m	5x5	T1 N0 M0	I	None
26	c.104T>A	*CTNNB1*	+	74	F	Ɨ 11y/8 m	†11y/8 m	16x15x12	T4 N0 M0	III	Cortisol+Androgen
27	No		-	67	M	Ɨ 9 y/6 m	†1y/7m	25x17x11	T3 N0 M0	III	None
28	c.136C>A	*CTNNB1*	+	60	F	Ɨ 11 y/8 m	†11 y/8	7.5x7.5	T2 N0 M0	II	Cortisol+Androgen
29	No		+	34	M	14 y/3 m	14 y/3 m	15x15	T2 N0 M0	II	None
30	No		-	62	F	Ɨ 13 y/2 m	†4y/9m	18x18x10	T2 N0 M0	II	None
31	c.646C>T	*ZNRF3*	-	62	M	Ɨ 9 y/7 m	†4y/5m	15x13x9	T2 N0 M0	II	Cortisol
32	No		-	60	M	Ɨ 10 m	†1m	5.5x5.5x6	T4 N0 M0	III	Cortisol
33	No		-	15	F	9 y/3 m	9y/3m	9x8x6	T2 N0 M0	II	Androgen
34	No		-	58	M	7 y/8 m	7y/8m	10x8x6	T2 N0 M0	II	None
35	No		-	57	F	Ɨ 3 m	†2m	19x9x10	T2 N0 M0	II	Cortisol+Androgen
37	c.-48-5464_241-51del	*CTNNB1*	+	78	M	Ɨ 4 m	-	9x9x10	T2 N0 M1	IV	Cortisol+Aldosterone
38	No		-	75	M	6y/7m	6y/7m	30x20x8	T2 N0 M0	II	Cortisol
40	c.-48-	*CTNNB1*	+	38	F	Ɨ 13 m	†3m	12x8x5	T4 N0 M0	III	None
41	No		-	64	F	5 y/2 m	5y/2m	11x9x8	T4 N0 M0	III	Cortisol+Androgen
42	No		+	28	M	Ɨ 4 m	-	10x10x6.5	T4 N0 M1	IV	Cortisol+Aldosteron
43	c.109C>G	*CTNNB1*	+	3	M	4 y/8 m	4y/8m	7x4.5x4.5	NA	III	Cortisol+Androgen
44	No		+	67	F	5 y/1 m	†3m	13x10.5x7	NA	III	None
46	c.105G>A	*CTNNB1*	+	67	F	Ɨ 1 y	†1m	8.5x5x5	T3 N1 M0	III	Cortisol
47	No		+	50	F	Ɨ 9 m	†3m	8x8x6	T4 N0 M0	IV	Cortisol
48	No		+	6	F	2 y/9 m	2y/9m	9x8x12	T3 N0 M0	II	Cortisol+Androgen
49	No		+	63	M	2 y/6 m	2 y/6 m	26x15x14	T3 N0 M0	III	Androgen
50	No		-	1	F	2 y/4 m	2y/4m	6x8x6.5	T4 N0 M0	III	Androgen
51	c.4391_4394del	*APC*	+	8	M	2 y/1 m	†1y/11m	11x8.5x8	T2 N0 M0	II	Cortisol
52	No		-	14	F	7m	7m	5x5x4	T1 N0 M0	I	Cortisol+Androgen
53	c.134C>T	*CTNNB1*	+	53	F	Ɨ 1 m	†1m	10x6x4	T4 N0 M0	III	Cortisol
54	No		+	43	F	1 y/8 m	†2m	9x8x7	T3 N1 M0	III	Cortisol
55	No		-	43	M	1 y/2 m	1y	19x14x12	T3 N0 M0	III	Cortisol

Ɨ : Deceased; †:Recurrence; F/M: Female/male; NA: Not Available; TNM : Tumour Node Metastasis

**Supplementary Table S8**						
		Age at operation	tumour size	Female;male	Cortisol producing	ENSAT stage
		(median in yrs)	(median length in cm)			III+IV; I+II
*APC/CTNNB1/ZNRF3*alterations	n= 22	52.5 (n=22)	10 (n= 22)	14;8 (n=22)	15 (n=22)	14;8 (n=22)
APC/CTNNB1/ZNRF3 wildtype	n= 30	46 (n=29)	10 (n=29)	17;12 (n=29)	14 (n=29)	15;14 (n=29)
				χ^2=0.005 p=0.9412	χ^2=1.291 p=0.2559	χ^2=0.320 p=0.5719
Nuclear accumulation	n=25	51.5 (n=24)	10 (n=24)	15;9 (n=24)	14 (n=24)	17;7 (n=24)
No nuclear accumulation	n=24	52.5 (n=24)	10 (n=24)	14;10 (n=24)	13 (n=24)	11;13 (n=24)
		p=0.5159	p=0.8097	χ^2=0.087 p=0.7679	χ^2=0.085 p=0.7711	χ^2=2.143 p=0.1432
Active β- catenin expression	n=30	46 (n=29)	9 (n=29)	18;11 (n=29)	16 (n=29)	16;13 (n=29)
lack of active β- catenin expression	n=16	58 (n=16)	10 ((n=16)	9;7 (n=16)	9 (n=16)	8;16 (n=16)
		p=0.3744	p=0.5059	χ^2=0.004 p=0.9493	χ^2=0.005 p=0.0.9445	χ^2=1.723 p=0.1893
Cohort	n=52	52 (n=51)	10 (n=51)	31;20 (n=51)	29 (n=51)	29;22 (n=51)

Supplementary Table S9. Cox regression analysis for overall survival				
**Univariate analysis**				
	Cohort (n=51)		Cohort – childhood ACC (n=46)	
	HR (95% CI)	P value	HR (95% CI)	P value
Age at operation	1.012 (0.994 – 1.032)	0.203	1.006 (0.986 – 1.,027	0.578
Cortisol production	1.763 (0.873 – 3.559)	0.114	1.890 (0.939– 3.806)	0.074
ENSAT staging (I/II vs II/IV)	4.076 (1.798 – 9.244)	0.001	4.160 (1.832 – 9.448)	0.001
Gender	1.037 (0.510 – 2.108)	0.919	0.993 (0.491 – 2.025)	0.993
Size	1.009 (0.954 – 1.068)	0.752	1.003 (0.948 -1.062)	0.907
APC/CTNNB1/ZNRF3 alteration	2.493 (1.211-5.131)	0.013	2.481 (1.206-5.107)	0.014
**Multuvariate analysis**				
	Cohort (n=51)		Cohort – childhood ACC (n=46)	
	HR (95% CI)	P value	HR (95% CI)	P value
Grading	3.480 (1.528-7.927)	0.003	3.604 (1.589-8.174)	0.002
APC/CTNNB1/ZNRF3 alteration	1.976 (0.949-4.113)	0.069	2.028 (0.979-4.201)	0.057

**Supplementary Table S10**		
CTNNB1	**DNA primers**	
	Exon 3	
	Fw primer	CAATGGGTCATATCACAGATTCTT
	Rw primer	CTGTGTAGATGGGATCTGC
	Exon 5	
	Fw primer	GCTCAAGGGGAGTAGTTTCAGA
	Rw primer	TCCACTGGTGAACTGGGAAG
	**cDNA Primers**	
	Fw primer	GAAGGTCTGAGGAGCAGCTTC
	Rw primer	ACAACACAGAATCCACTGGTG
	**DNA primers**	
ZNRF3	Exon 2	GGGTTGAAAAACTGTCCCCA
		CCACCTCATGCAACTTCAGC
	Exon 3	GATTGCCAAGGCCAACTTT
		GGGACAAGCCAAGCTCACTA
	Exon 4	CTGGAGGATTCCAGACAGGT
		GGGTATGCCAGCATTTCAGA
	Exon 5	GATAGCCCATGTGCCGCTTA
		GCTGTGAGGCTCCATGTTGC
	Exon 6	GTCTGCCTGTCCCAGTGAAT
		CCTTCTAGGTCTTGGGCACC
	Exon 7	GCTGTGCAGAACTCCTTGG
		GAGTGTTCCCAAGCCTGCG
	Exon 8a	GCCTCTGACACCAGTATGCT
		CTACCACAGAGTCACTGGAG
	Exon 8b	GCTTCAGCTGCTATCACGGC
		GTCCTGGGCAAATGGTCGG
	Exon 8c	TGGCAGCAGCACCTTGTTC
		GGCCAGGCTGAGGAGTAACC
	Exon 9	TTGCCTGGTCCCATGTGTGG
		TGGGTTGACAAGGAGGGCTC

References

1 Xing, Y., Clements, W. K., Kimelman, D. & Xu, W. Crystal structure of a beta-catenin/axin complex suggests a mechanism for the beta-catenin destruction complex. *Genes & development* **17**, 2753-2764, doi:10.1101/gad.1142603 (2003).

2 Gottardi, C. J. & Peifer, M. Terminal regions of beta-catenin come into view. *Structure* **16**, 336-338, doi:10.1016/j.str.2008.02.005 (2008).
